# Identification of Epithelial Mesenchymal Transition-Related lncRNAs Associated with Prognosis and Tumor Immune Microenvironment of Hepatocellular Carcinoma

**DOI:** 10.1155/2022/6335155

**Published:** 2022-01-15

**Authors:** Yongjie Zhou, Liangwen Wang, Wen Zhang, Jingqin Ma, Zihan Zhang, Minjie Yang, Jiaze Yu, Jianjun Luo, Zhiping Yan

**Affiliations:** ^1^Department of Interventional Radiology, Zhongshan Hospital, Fudan University, Shanghai, China; ^2^Shanghai Institution of Medical Imaging, Shanghai, China; ^3^National Clinical Research Center for Interventional Medicine, Zhongshan Hospital, Fudan University, Shanghai, China; ^4^Center for Tumor Diagnosis and Therapy, Jinshan Hospital, Fudan University, Shanghai, China

## Abstract

**Purpose:**

The long noncoding RNAs (lncRNAs) play the important role in tumor occurrence and progression, and the epithelial to mesenchymal transition (EMT) is the critical process for tumor migration. However, the role of EMT-related lncRNA in hepatocellular carcinoma (HCC) has not been elucidated.

**Methods:**

In this study, we selected the EMT-related lncRNAs in HCC by using data from The Cancer Genome Atlas database (TCGA). Two prognostic models of the overall survival (OS) and relapse-free survival (RFS) were constructed and validated through Cox regression model, Kaplan-Meier analysis, and the receiver-operating characteristic (ROC) curves. The unsupervised clustering analysis was utilized to investigate the association between EMT-lncRNAs with tumor immune microenvironment. ESTIMATE algorithm and gene set enrichment analysis (GSEA) were used to estimate tumor microenvironment and associated KEGG pathways.

**Results:**

Two EMT-related lncRNA prognostic models of OS and RFS were constructed. Kaplan-Meier curves showed the dismal prognosis of OS and RFS in the group with high-risk score. The ROC curves and AUC values in two prognostic models indicated the discriminative value in the training set and validation set. Patients with HCC were clustered into two subgroups according the unsupervised clustering analysis. Lnc-CCNY-1 was selected as the key lncRNA. GSVA analysis showed that lnc-CCNY-1 was negatively associated with peroxisome proliferator-activated receptor (PPAR) signaling pathway and positively correlated with CELL cycle pathway.

**Conclusion:**

Two EMT-related lncRNA prognostic models of OS and RFS were constructed to discriminate patients and predict prognosis of HCC. EMT-related lncRNAs may play a role on prognosis of HCC by influencing the immune microenvironment. Lnc-CCNY-1 was selected as the key EMT-related lncRNA for further exploration.

## 1. Introduction

Hepatocellular carcinoma (HCC) is the most common cancer and the third leading cause of cancer-related deaths worldwide [1]. Despite great advance had made in the treatment modality for HCC, the prognosis of HCC remains dismal with 1 − year survival < 50% due to high recurrence rate [2]. The frequent methods for classification and prognosis prediction of patients with HCC were the Barcelona-Clinic Liver Cancer (BCLC) staging system [3]. However, it could not precisely predict prognosis of patients with HCC due to high heterogeneous of HCC. It is necessary to construct a novel method to discriminate patients and predict prognosis.

The epithelial to mesenchymal transition (EMT) is a multistep biological process, which is characterized by epithelial cell lose polarity and connection to the basement membrane and gains the ability of invasion, migration, and resistance to apoptosis. EMT has been reported to play a crucial role in early steps of metastasis in carcinoma progression [4]. Several studies had reported that various genes regulate EMT to affect tumor process of invasion, metastasis and proliferation in HCC [5–8].

Due to the development of next-generation sequencing technology, the tumor gene characteristics received more attention. LncRNA characterized by greater than 200 nt in length was a form of noncoding RNAs. It was reported that lncRNAs are incorporated in several pathophysiological processes of tumor progression [9–13]. Several studies had demonstrated that lncRNAs were associated with prognosis and HCC proliferation, invasion, and metastasis [14–16]. Therefore, we speculated that EMT-related lncRNA may serve a promising target for predicting prognosis of patients with HCC.

Immunotherapy has made great advances and has been emerged as an effective treatment in HCC and other malignant tumors [17–20]. However, only a small portion of patients with HCC could obtain favorable prognosis from immunotherapy. It has been demonstrated that tumor microenvironment (TME) acted a critical role in tumor progression and affected the effect of immunotherapy. TME defined as the environment around a tumor included the surrounding blood vessels, immune cells, adipocytes, mesenchymal stem cells, and the extracellular matrix (ECM) [21, 22]. Several studies had reported that CD8+, CD4+cells, regulatory T cells, and dendritic cells (DCs) which were located in TME were associated with the efficacy of immunotherapy by influence immune checkpoint inhibitors (ICIs) [23, 24]. However, the identification of Tertiary Lymphoid Structures (TLS) cells is not sufficient to characterize the complex tumor immune milieu. Then, it is worth to investigate landscape of immune cells infiltrating the TME of HCC.

In this study, we identified differentially expressed EMT-related lncRNAs by comparing the tumor samples and the normal samples from The Cancer Genome Atlas database (TCGA) database. Then, the prognostic EMT-related lncRNA models were constructed and validated. The key EMT-related lncRNA which was simultaneously related to overall survival (OS) and disease-free survival (RFS) was selected for further investigation. ESTIMATE and CIBERSORT algorithms were performed to explore the intratumoral immune landscape.

## 2. Materials and Methods

### 2.1. Patient Datasets

The gene expression profile of HCC patients was downloaded from TCGA-LIHC database, and the clinical information of these patients was retrieved from cBioportal database. We selected a list of lncRNA by using the gene annotation in the GENCODE project. All data were downloaded from public database, so ethical approval and informed consent were waived.

### 2.2. Differentially Expressed EMT-Related lncRNA

The EMT-related gene profile from EMT gene database (http://dbemt.bioinfo-minzhao.org/download.cgi) was downloaded. We performed Pearson correlation analysis to select EMT-related lncRNAs, when the correlation coefficient ∣*r* | >0.4 and *p* < 0.05. We obtained the differentially expressed EMT-related lncRNAs by comparing the tumor samples and the normal samples using the “limma” package in the R software, with ∣logFC | >1 and a false discovery rate < 0.05.

### 2.3. Construction and Validation of Prognostic EMT-Related lncRNA Signature

HCC patients were randomly divided into the training set and the validation set with ratio 7 : 3. HCC patients in the training set were used to construct the prognostic signature, while HCC patients in the validation set were performed to verify the established signature. The univariate Cox proportional regression analysis was performed to screen preliminary EMT-related lncRNAs associated with OS and RFS, which were further analyzed using the multivariate Cox proportional hazard regression model. Finally, the prognostic EMT-LncRNAs were identified to calculate risk score. The risk score of each patient with HCC in both training and validation sets was calculated as below formula: Risk score = *β*lncRNA1 × lncRNA1 Expression + *β*lncRNA2 × lncRNA2 Expression + *β*lncRNA3 × lncRNA3 Expression + ⋯+*β*lncRNAn × lncRNAn Expression. Here, “*β*” was the regression coefficient which was generated from the multivariate Cox proportional hazard regression analysis.

Based on the median risk score, HCC patient in both sets were allocated into high- or low-risk groups. The Kaplan-Meier survival curves were performed to compare prognosis of two groups, and the receiver-operating characteristic (ROC) curves at 1, 3, and 5 years were utilized to evaluate the predictive ability of this model. To further analyze prognostic value in different stages of HCC patients, we performed the ROC curves at 1, 3, and 5 years in four stages.

### 2.4. mRNA-lncRNA Network

We performed the Pearson correlation analysis to explore the interaction between mRNA and EMT-related lncRNA. It was considered significant association, with the criterion of correlation coefficient ∣*R* | >0.4 and *p* < 0.005. Cytoscape was utilized to visualize the coexpression network.

### 2.5. Correlation of EMT-Related lncRNAs with Immune Tumor Microenvironment

We utilized the unsupervised consensus approach to generate two clusters of HCC cohort through “Consensus Cluster Plus” package, to investigate the association between EMT-lncRNAs with tumor immune microenvironment. ESTIMATE algorithm [25] was used to estimate tumor purity and the extent and level of infiltrating cells (stromal cell, immune cell, and ESTIMATE score), which represented the characteristics of tumor immune microenvironment. Subsequently, the Single Sample Gene Set Enrichment Analysis (ssGSEA) algorithm was used to elucidate the enrichment of 29 immune function-related gene sets, and CIBERSORT algorithm was performed to quantify the fraction of 22 types of immune cells in each sample [26]. We correlated clusters with survival prognosis, clinical features, and immune infiltration.

### 2.6. A Key EMT-Related lncRNA in HCC

Based on the multivariate Cox proportional hazard regression analysis, differentially expressed EMT-related lncRNAs were identified, including for OS signature and for RFS signature. Among them, EMT-related lncRNA lnc-CCNY-1 was both associated with OS and RFS. The Kaplan-Meier survival curves and log-rank test were performed for lncRNAs for further research. The EMT-related lncRNAs which showed the more relevant to prognosis with prognosis of HCC was identified.

Gene Set Variation Analysis (GSVA) was utilized to characterize pathways or signature summaries in samples of expression datasets. Multivariate Cox proportional hazard regression analysis was used to select pathways associated with prognosis, and Pearson correlation analysis was used to investigate the association of prognosis-related pathways with immune microenvironment.

## 3. Results

### 3.1. Identification of Differentially Expressed EMT-Related lncRNAs

**Figure 1:**
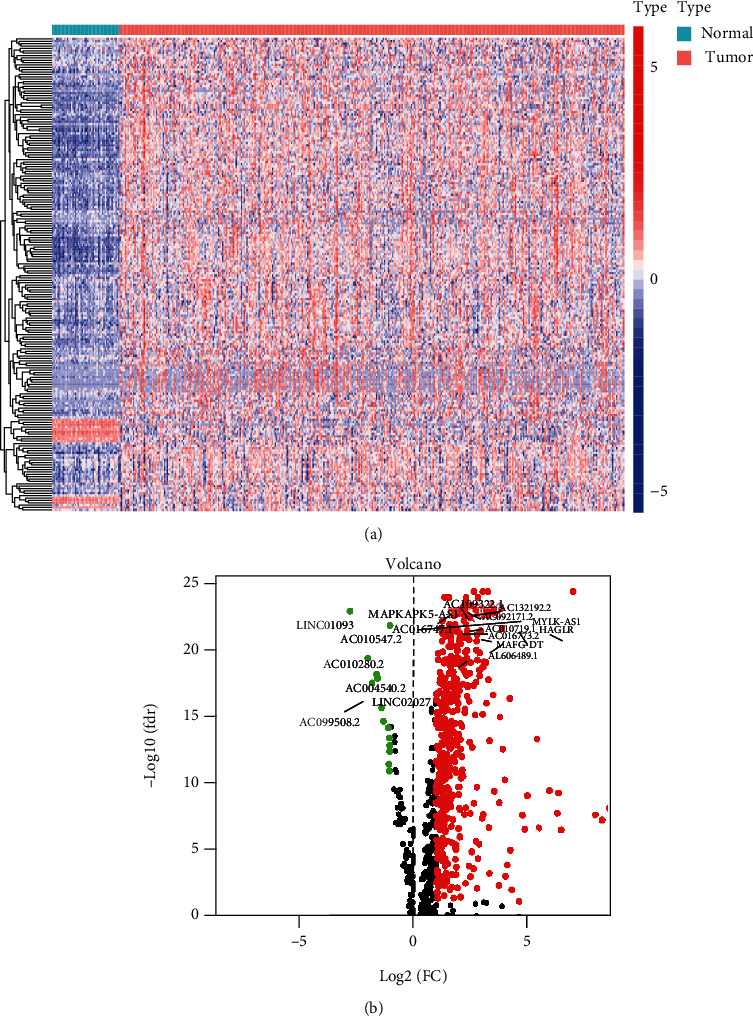
Identification of differentially expressed EMT-related lncRNAs. The heatmap (a) and the volcano plot (b) showed the differentially expressed EMT-related lncRNAs between HCC tumor tissues and adjacent normal tissues.

Overall, 374 tumor samples and 50 adjacent normal samples from TCGA-LIHC database were incorporated in our research. We identified EMT-related lncRNAs by using the TCGA database and EMT gene database. Then, the differentially expressed EMT-related lncRNAs were selected by the comparison of tumor tissues with adjacent normal tissues, which included 478 upregulated lncRNAs and 14 downregulated lncRNAs (Supplementary Table S1). The heatmap and volcano plot were showed characteristics of these differentially expressed EMT-related lncRNAs (Figures 1(a) and 1(b)).

### 3.2. Construction and Validation of Prognostic Models

Based on the criteria, we obtained 377 HCC patients with clinical information from TCGA database. The clinicopathological characteristics of these patients were presented in Table 1.

These 377 HCC patients with HCC were randomly divided into the training set (*n* = 264) and validation set (*n* = 113) with the ratio of 7 : 3. We performed the univariate Cox regression analysis to select the EMT-related lncRNAs, which were associated with prognosis in the training set. 492 lncRNAs were determined for OS signature, and 480 lncRNAs were selected for RFS signature (Supplementary Tables S2, S3). We further conducted multivariate Cox regression to construct the prognostic model, and nine EMT-related lncRNAs for OS prognosis and three EMT-related lncRNAs for RFS prognosis were identified (Tables 2 and 3). The risk scores were calculated as follows: OS signature = 1.57987 × lnc − CCNY − 1 expression + 1.04763 × AC022424.1 expression + 1.03481 × AC079305.2 expression + 1.23469 × AC068506.1 expression + 1.51943 × AC026369.2 expression + 1.04672 × ELFN1 − AS1 expression + 1.15117 × AC009005.1 expression + 1.26892 × AC068580.1 expression + 1.08326 × AL049840.6 expression, RFS signature = 1.09467 × ZFPM2 − AS1 expression + 1.33526 × lnc − CCNY − 1 expression + 1.10833 × AC005332.3 expression.

**Figure 2:**
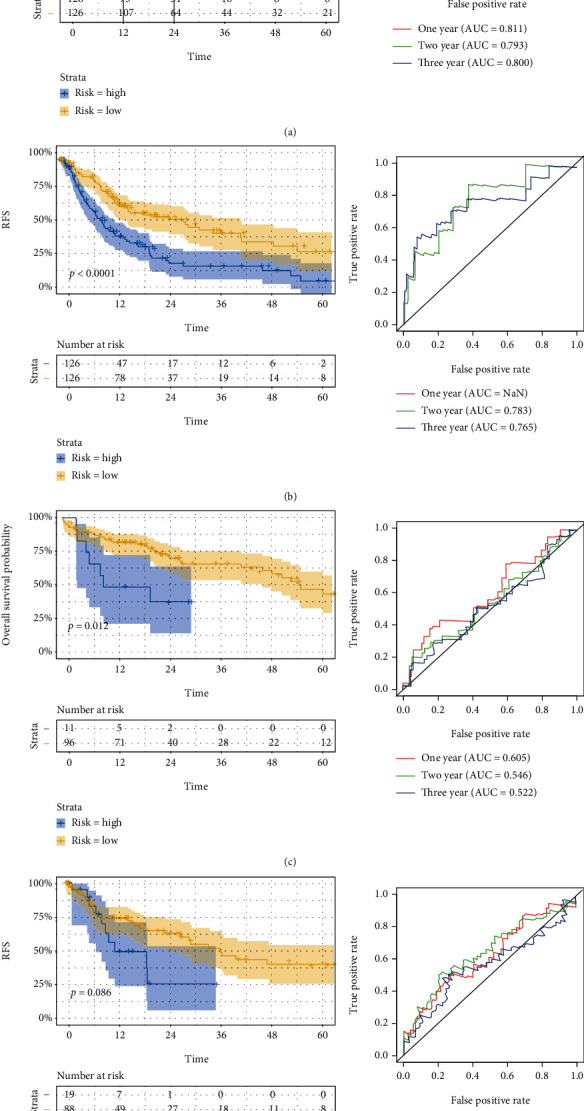
Validation of two prognostic models. Kaplan-Meier survival curve and ROC curve for OS and RFS in the training set (a, b) and the validation set (c, d).

Based on the median cut-off value, patients were divided into the high-risk group and low-risk group both in the training set and validation set. In the training set, Kaplan-Meier curves showed the favorable prognosis of OS and RFS in the low-risk group (Figures 2(a) and 2(b)). Besides, ROC curves were performed for prediction for OS and RFS by using above prognostic model, and the area under the curve (AUC) values for OS at 1, 3, and 5 years were 0.605, 0.546, and 0.522, and AUC values for RFS at 1, 3, and 5 years were 0.621, 0.646, and 0.565, respectively. The ROC curves and AUC values in both prognostic models indicated the discriminative value in both prognostic models. In line with above results, the Kaplan-Meier curves from the validation set also showed the prognosis of OS and RFS was dismal in the high-risk group. The satisfactory AUC values also verified the predictive ability of two signatures (Figures 2(c) and 2(d)).

### 3.3. Establishment of mRNA-lncRNA Networks

**Figure 3:**
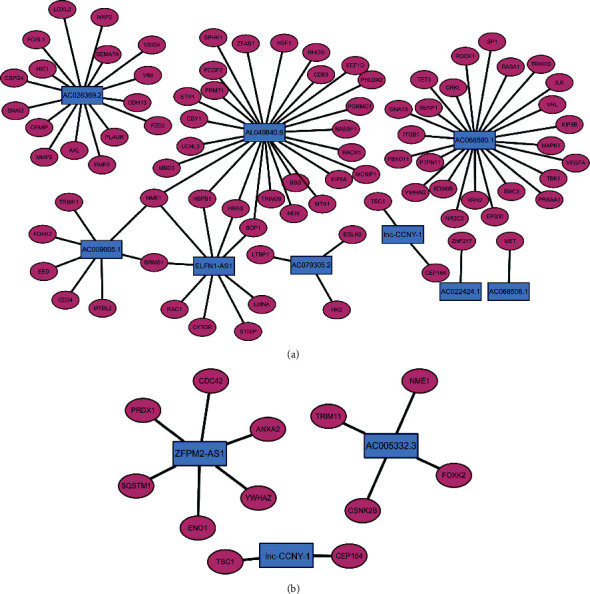
mRNA-lncRNA networks associated with OS signature (a) and RFS signature (b).

The Pearson correlation analysis was utilized to established the mRNA-lncRNA coexpression network in order to explore the regulatory mechanism between lncRNA and mRNA. The Figure 3(a) showed the network between mRNAs and nine EMT-related lncRNAs associated with OS prognosis, and Figure 3(b) displayed the network between mRNAs with three EMT-related lncRNAs related with RFS signature. When we selected the lnc-CCNY-1 which was simultaneously associated with OS and RFS into further analysis, TSC1 and CEP164 were obtained as the target genes. These genes were reserved for future research.

### 3.4. Characteristics of Gene Set Variation Analysis and Immune Cell Infiltration

**Figure 4:**
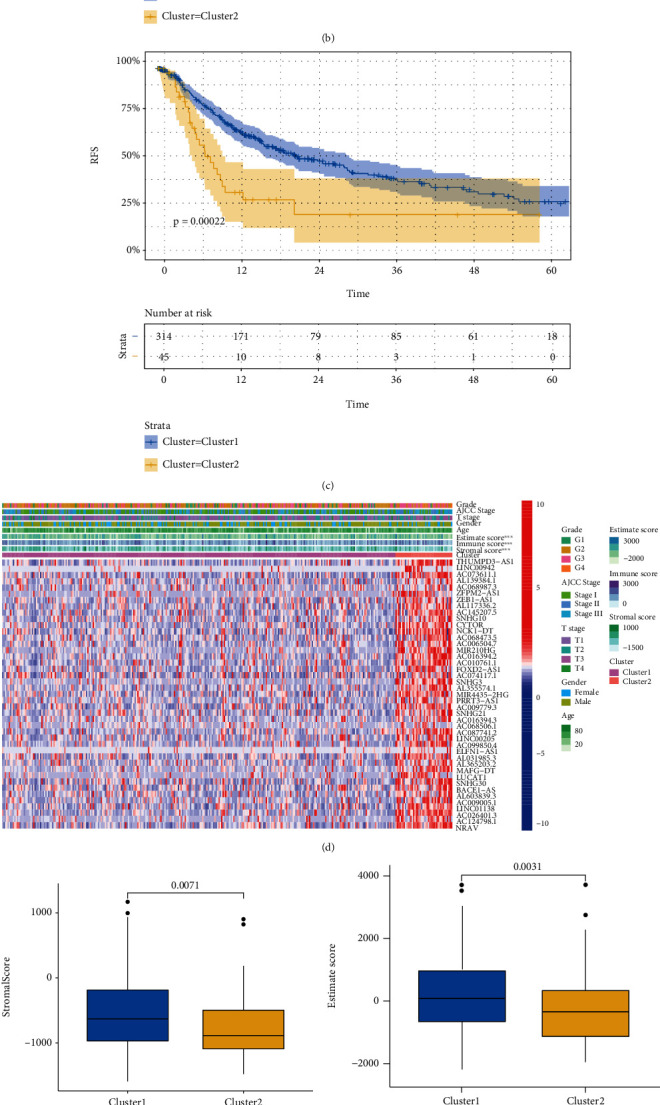
The association of prognosis and immune landscape with EMT-related lncRNAs. Patients with HCC were grouped into two clusters according to the consensus clustering matrix (*k* = 2) (a). Kaplan-Meier survival curve for OS (b) and RFS (c) between two clusters. The heatmap of clinicopathological characters, immune microenvironment, and EMT-related lncRNA expression between two clusters (d). Comparison of stromal score, ESTIMATE score, and immune score between the two clusters (e–g). Difference of infiltrating immune cell between two clusters (h).

Based on the similarity of expression of EMT-related lncRNAs, the *k* = 2 was finally selected with optimal clustering stability (Figure 4(a)). A total of 377 patients with HCC were clustered into two subgroups, in cluster C1 and in cluster C2. The Kaplan-Meier curves showed that OS (Figure 4(b)) and RFS (Figure 4(c)) of cluster C1 were longer than those of cluster C2 (*p* < 0.0001, *p* = 0.00022, respectively). As shown in Figures 4(d), 4(e), and 4(g), cluster C1 had higher stromal score, ESTIMATE score, and immune score than cluster C2. The discrepancy of composition of 22 immune cells between two clusters showed that higher T regulatory cells (Tregs) and lower macrophages M2 were shown in cluster C1, as presented in Figure 4(h).

### 3.5. Lnc-CCNY-1 Is the Key EMT-Related lncRNA in HCC

**Figure 5:**
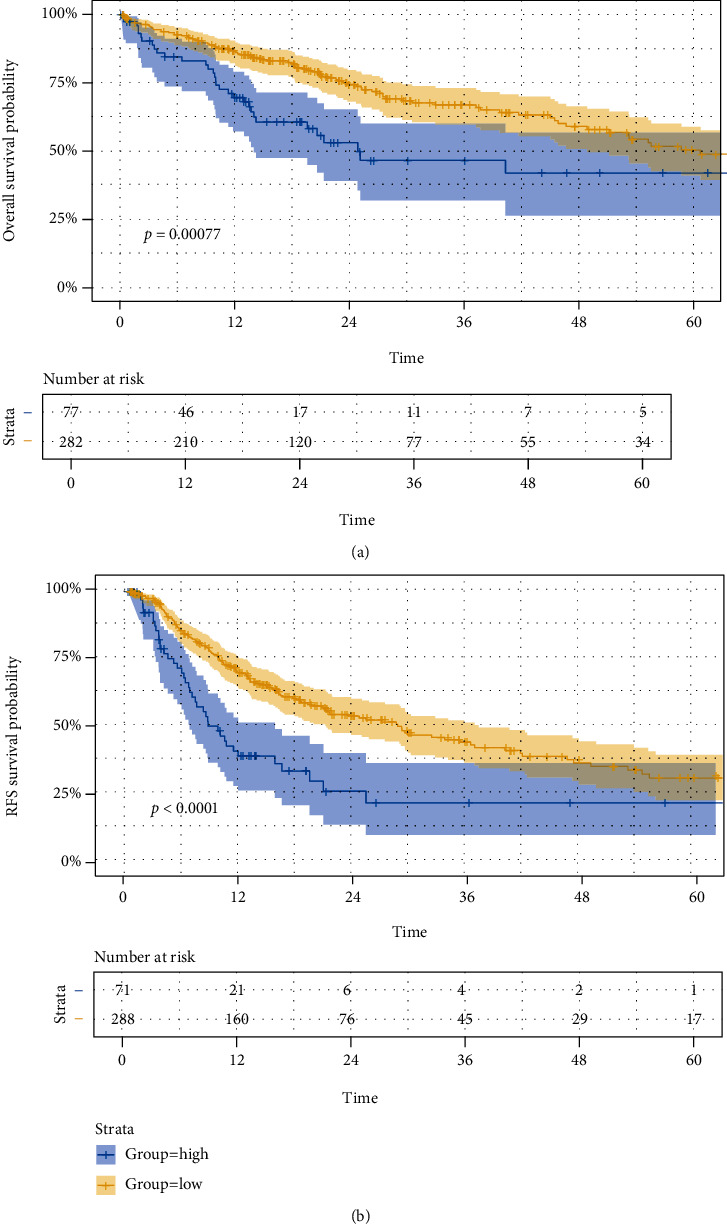
Kaplan-Meier survival curve for OS and RFS, according to the expression of Lnc-CCNY-1.

**Figure 6:**
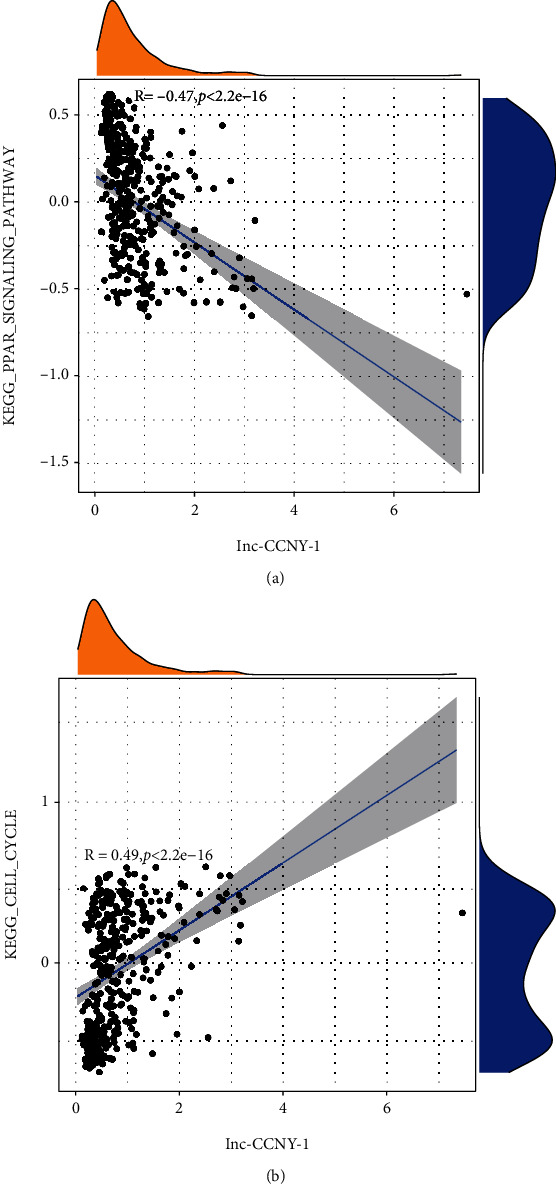
The relationship of Lnc-CCNY-1 and pathways.

To further investigate the biomarkers for prognosis and tumorigenesis in HCC, we selected the tumorigenesis lncRNA which was simultaneously incorporated into OS prognostic model and RFS prognostic model. The Kaplan-Meier curves showed that patients with high expression of lnc-CCNY-1 had the unfavorable prognosis in both OS and RFS (Figures 5(a) and 5(b)). GSVA analysis was utilized to further explore the biological function of the key EMT-related lncRNAs. The further analysis showed that lnc-CCNY-1 lncRNA was significantly positive associated with cell cycle pathway and negatively associated with peroxisome proliferator-activated receptor (PPAR) signaling pathway, as shown in Figures 6(a) and 6(b).

**Figure 7:**
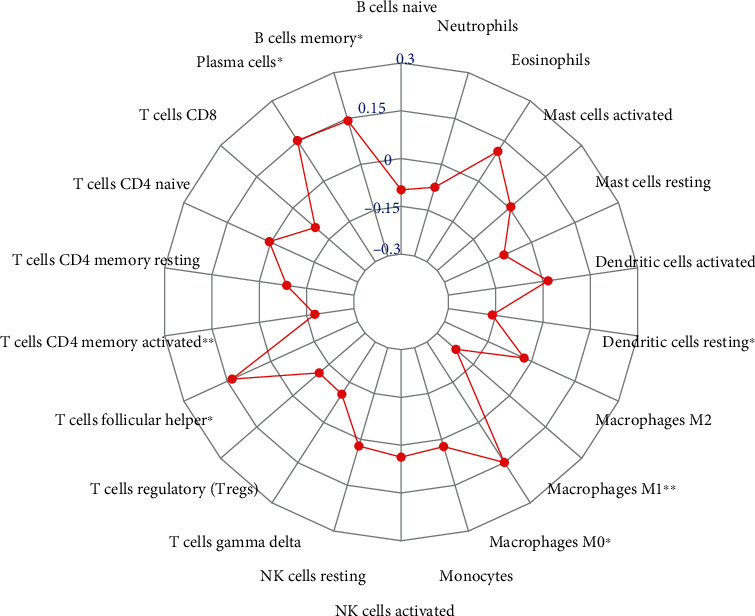
Correlation between immune cells and the expression of Lnc-CCNY-1 (^∗^ indicated *p* < 0.05, ^∗∗^ indicated *p* < 0.01).

To further investigate the regulatory mechanism of lnc-CCNY-1, the radar chart was drawn to show the correlation with immune cells (Figure 7). The expression of lnc-CCNY-1 was positively associated with plasma cells, macrophages M0, and T cell follicular helper and negatively correlated with CD4 memory-activated macrophages M1.

## 4. Discussion

The pathogenesis of HCC was very complicated because occurrence resulted from several factors, including internal gene factors, external environment, and progress of chronic liver disease [24]. Besides, preliminary investigation also demonstrated that the occurrence and progression of HCC were significantly associated with cell cycle, apoptosis, and energy metabolism abnormalities [27, 28]. So, it is worth to understand the tumorigenesis and evolution mechanism of HCC due to the dismal prognosis in clinical. The increasing studies showed that EMT and lncRNAs were incorporated into the progression of HCC [29–32]. In our study, two EMT-related lncRNA prognostic models of OS and RFS were constructed. Kaplan-Meier curves showed the favorable prognosis of OS and RFS in the low-risk group. The ROC curves and AUC values in two prognostic models indicated the discriminative value in the training set and validation set.

EMT is a cellular program which played a crucial role for embryogenesis, wound healing, and malignant progression [33]. In this course, the stone epithelial appearance of epithelial cells was lost to adopt a spindle shaped, mesenchymal morphology. During the course of tumor progression, EMT confers on individual carcinoma cell multiple traits in many cancers [34–37]. Previous studies had showed that genes influenced the proliferation, invasion, and metastasis of HCC by regulating EMT procedure [38, 39]. For instance, Meng et al. [6] found that USP5 could accelerate HCC cell proliferation, metastasis, and invasion through coordinate EMT program. Increasing investigations had demonstrated that lncRNAs showed the crucial role in diverse physiological and pathological processes. It was reported [40] that lncRNA MIAT accelerated proliferation and invasion of HCC cells by regulating miR-214. The study performed by Pan et al. demonstrated that lncRNA-PDPK2P promoted HCC progression through the PDK1/AKT/Caspase 3 pathway.

EMT-related lncRNAs were identified by using TCGA database and EMT gene base. Then, we selected the differentially expressed EMT-related lncRNAs by comparing the tumor tissues and adjacent normal tissues. Finally, nine lncRNAs were significantly associated with OS, and three lncRNAs were correlated with RFS. The risk scores of each patient were calculated by the coefficient and expression of aforementioned lncRNAs and further divided the patients with HCC into the high-risk group and low-risk group. In training set, Kaplan-Meier curves showed the favorable prognosis of OS and RFS in the low-risk group. The ROC curves and AUC values in both prognostic models indicated the discriminative value in both prognostic models. In line with the conclusion in the training set, the same results of Kaplan-Meier curves and high accuracy of prognostic models were shown in the validation set.

In recent years, TME had received more attention due the immunotherapy had showed curative effect in cancer management. TME acted a critical role in tumor progression and affected the effect of immunotherapy. To investigate the association between EMT-related lncRNAs with TME, “Consensus Cluster Plus” package was used to generate two clusters of HCC cohort. The Kaplan-Meier curves in our study showed that OS and RFS of cluster C1 were longer than those of cluster C2. The differences between two clusters in terms of stromal score, ESTIMATE score and immune score, indicate that EMT-related lncRNAs may affect tumor progression by regulating TME. A previous study performed by Peng et al. [41] demonstrated that LncRNA MIAT was significantly associated with immune escape of HCC and altered the immune cell infiltration. Ji et al. [42] reported that Lnc-Tim3 was significant with CD8+T exhaustion and the survival of the exhausted CD8+T cells. Our results showed that the higher expression of Tregs and lower expression of macrophages M2 were shown in cluster C1 which had favorable prognosis, with the comparison with cluster 2. Tregs which mainly derived from peripheral blood T lymphocytes were significantly correlated with HCC invasiveness and played important roles in hampering effect of antitumor responses in HCC [43–45]. It had been reported that activated Tregs prohibit different types of immune cells by contact-dependent interactions and further contribute to immune dysfunction in HCC [46]. M2-polarized macrophages characterized as tumor-associated macrophages (TAMs) were the important composition of tumor-infiltrating immune cells. The study by Yao et al. reported that M2-polarized macrophages accelerated the migration and invasion of HCC cells via EMT process [47]. The regulatory mechanism of TME had not been elucidated and is worthy to investigate in future research.

To further investigate the biomarkers for prognosis and tumorigenesis in HCC, lnc-CCNY-1 which was simultaneously incorporated into OS prognostic model and RFS prognostic model was identified in our research. The expression of lnc-CCNY-1 was negatively with progression in terms for OS and RFS, indicating lnc-CCNY-1 may promote tumor progression of HCC. GSVA analysis showed that lnc-CCNY-1 was negatively associated with PPAR signaling pathway and positively correlated with CELL cycle pathway. Previous studies had reported that PPAR signaling pathway was downregulated in colorectal cancer and non-small-cell lung cancer [48, 49]. However, there was no investigation about PPAR signaling pathway in HCC, which is reserved for further research. Cell cycle pathway was a critical mechanism, by which many genes could promote HCC cell proliferation [50–52]. The reasonable explanation was that lnc-CCNY-1 promote tumor progression of HCC by downregulating PPAR signaling pathway and upregulating cell cycle.

It was noted that our study also included several limitations. Firstly, the EMT-related lncRNA prognostic model was generated by bioinformatic approaches, which is not validated in our registers. Besides, lnc-CCNY-1 was identified as the key EMT-related lncRNA, which was not verified in in vivo or further in vitro experiment in this study. We plan to further investigate the mechanism of lnc-CCNY-1 for HCC in next research.

## 5. Conclusion

Two EMT-related lncRNA prognostic models of OS and RFS were constructed to discriminate patients and predict prognosis of HCC. EMT-related lncRNAs may play a role on prognosis of HCC by influencing the immune microenvironment. Lnc-CCNY-1 was selected as the key EMT-related lncRNA for further research.

## Figures and Tables

**Table 1:** The clinicopathological characteristics of patients with HCC.

Characteristics	No. (%)
Gender	
Male	255 (67.6)
Female	122 (32.3)
Age	
≤65	236 (62.5)
>65	141 (37.5)
Grade	
G1-G2	236 (62.6)
G3-G4	137 (36.3)
Unknown	4 (1.1)
Stage	
Stage I-II	262 (69.5)
Stage III-IV	92 (24.4)
Unknown	23 (6.1)
T stage	
T1-T2	279 (74.0)
T3-T4	92 (24.4)
Unknown	6 (1.6)
N stage	
N0	255 (67.6)
N1	118 (31.3)
Unknown	4 (1.1)
M stage	
M0	272 (72.1)
M1	101 (26.8)
Unknown	4 (1.1)

**Table 2:** EMT-related lncRNAs associated with overall survival (OS).

ID	Coef	HR	HR.95L	HR.95H	*p* value
lnc-CCNY-1	0.457342603	1.57987006	1.250877801	1.995390281	0.00012358
AC022424.1	0.046529762	1.047629258	1.009914891	1.086752034	0.012868687
AC079305.2	0.034220542	1.034812801	1.012748546	1.05735776	0.001858415
AC068506.1	0.210820365	1.234690542	1.104272923	1.380510834	0.000214405
AC026369.2	0.418336152	1.519431348	1.230999207	1.875445255	9.82E-05
ELFN1-AS1	0.045657717	1.046716077	1.004076559	1.091166341	0.03142199
AC009005.1	0.140782576	1.151174328	1.024057994	1.294069615	0.018364991
AC068580.1	0.238162915	1.268915901	1.066708751	1.509453787	0.007164066
AL049840.6	0.079975567	1.0832606	1.011253892	1.160394572	0.022676322

**Table 3:** EMT-related lncRNAs associated with relapse-free survival (RFS).

ID	Coef	HR	HR.95L	HR.95H	*p* value
ZFPM2-AS1	0.090455547	1.094672845	1.040883457	1.151241889	0.000433751
lnc-CCNY-1	0.289126912	1.335261178	1.04681349	1.703190139	0.019890581
AC005332.3	0.102852689	1.108328128	1.055492193	1.163808931	3.67E-05

## Data Availability

The data used to support the findings of this study are included within the article and the supplementary information files.

## Supplementary Materials

Supplementary 1 Supplementary Table 1: the differentially expressed EMT-related lncRNAs between tumor tissues with adjacent normal tissues.

Supplementary 2 Supplementary Table 2: 492 EMT-related lncRNAs associated with OS signature screened by the univariate Cox regression analysis.

Supplementary 3 Supplementary Table 3: 480 EMT-related lncRNAs associated with RFS signature screened by the univariate Cox regression analysis.
