# Supplementary material for: Identification of Epithelial Mesenchymal Transition-Related lncRNAs Associated with Prognosis and Tumor Immune Microenvironment of Hepatocellular Carcinoma
Source: Dis Markers. 2022 Jan 15;2022:6335155. doi: 10.1155/2022/6335155 (PMC8802097; doi:10.1155/2022/6335155)
Supplement: Supplementary 1 — Supplementary Table 1: the differentially expressed EMT-related lncRNAs between tumor tissues with adjacent normal tissues. [file 6335155.f1.pdf]

Gene	Conmean	Treatmean	LogFC	P-value	FDR
SNHG15	0.907477468	1.884031771	1.053889566	8.83E-06	1.22E-05
AL354733.3	0.26252006	0.553279036	1.075579593	0.001074475	0.00128661
MUC12-AS1	0.061443122	0.620857376	3.336938462	0.000339021	0.000420815
AC006042.1	0.15682245	0.648315566	2.047564105	4.47E-09	7.77E-09
VPS13B-DT	0.732605134	2.321344405	1.663852867	4.04E-15	1.29E-14
AP006621.2	0.246960162	1.609353969	2.704131436	1.54E-16	5.87E-16
THUMPD3-A!	0.417552991	1.434057606	1.780071775	6.85E-25	2.22E-23
AC025265.1	0.154723189	0.565949461	1.870983789	4.97E-12	1.15E-11
AC022613.1	1.232899851	3.891621353	1.658315732	8.41E-08	1.31E-07
AL451165.2	2.105298574	4.605082112	1.12920203	6.73E-12	1.54E-11
AL359513.1	0.120213997	0.575207539	2.258477702	4.98E-17	2.01E-16
LINC02428	10.75336732	4.042470966	-1.41147918	4.04E-17	1.66E-16
AC005586.1	0.291504936	0.867053709	1.572601052	8.31E-13	2.07E-12
MYLK-AS1	0.097408552	0.699673579	2.844561674	7.53E-27	9.41E-25
LINC00942	0.006149974	1.555603151	7.982678041	1.01E-08	1.70E-08
LINC00886	0.431780399	1.150869243	1.414354273	4.27E-05	5.66E-05
AL355353.2	1.183043683	2.465144557	1.059168904	0.000335526	0.00041717
ZNF529-AS1	0.29410918	0.914214179	1.636180378	1.19E-20	9.18E-20
ZNF213-AS1	0.499615116	1.26714217	1.342689369	9.14E-21	7.34E-20
LINC00896	0.094482152	0.652228846	2.787264514	7.72E-11	1.63E-10
AC073611.1	0.204748851	0.73300742	1.839972443	1.15E-18	5.83E-18
AL135905.1	1.398505436	3.803966156	1.443618548	4.80E-12	1.11E-11
TAT-AS1	0.427066045	0.881869218	1.046105521	0.009453748	0.010849994
DANCR	4.42437974	11.78658525	1.413598688	4.06E-09	7.10E-09
AL645933.3	0.712655894	1.569219832	1.13876993	1.98E-08	3.26E-08
SNHG12	0.473602196	1.811266972	1.935251534	7.10E-22	8.41E-21
LINC01139	0.035455534	0.881145753	4.635297951	0.04486599	0.049365824
AC093673.1	3.16342621	7.333990174	1.213110389	2.10E-11	4.63E-11
AC005586.2	0.25121224	0.582298627	1.212852456	1.09E-06	1.61E-06
LINC01942	0.68969465	1.450846176	1.072864887	2.74E-06	3.90E-06
LNCTAM34A	0.31955671	0.918124894	1.52261844	9.60E-15	2.94E-14
SNHG8	19.47856064	40.22251932	1.046116373	2.53E-11	5.53E-11
AL161669.3	0.361577441	1.433956836	1.987625024	5.16E-15	1.62E-14
TMPO-AS1	0.223927634	1.15424192	2.36584115	1.34E-23	2.70E-22
MAN1B1-DT	0.292326951	0.618133133	1.080334753	1.54E-09	2.80E-09
AL139384.1	0.539316853	1.44040913	1.417273626	1.21E-14	3.66E-14
AZIN1-AS1	0.249968357	0.644121722	1.365587867	4.11E-12	9.61E-12
AC112491.1	0.980105964	1.973377868	1.009657594	0.001643111	0.001942569
AC068987.3	0.268474978	1.605062914	2.579770308	5.60E-14	1.55E-13
AC010547.2	2.315825053	1.13197425	-1.03268513	5.12E-24	1.16E-22
AC016405.3	0.210645307	0.929251079	2.141252683	3.03E-12	7.17E-12
AL606489.1	0.055938465	0.834341378	3.898725219	8.23E-24	1.80E-22
ZSCAN16-AS	3.443201204	7.500916206	1.123316338	1.64E-12	3.99E-12

AC009407.1	3.991087935	10.44650534	1.388166428	3.63E-09	6.37E-09
AC090589.3	0.359481951	0.77462497	1.10757867	5.67E-08	9.04E-08
ZFPM2-AS1	0.053864775	1.493454266	4.793167127	1.20E-08	2.02E-08
AC012510.1	0.287810868	0.949093298	1.721428846	8.56E-17	3.36E-16
RAB30-DT	1.130121368	2.478681231	1.133095029	2.14E-18	1.04E-17
AC011468.1	0.290868174	1.169254251	2.00715132	8.44E-19	4.37E-18
LINC01671	0.162632608	0.682321854	2.068835879	0.021908058	0.024613571
AL355102.4	0.350581938	2.513657844	2.841964709	2.42E-05	3.27E-05
ZNF710-AS1	0.840688865	1.757579949	1.063946447	2.47E-05	3.31E-05
ASMTL-AS1	0.607834089	2.796766678	2.202010408	1.22E-20	9.30E-20
AC020765.2	0.257707804	0.923856849	1.8419331	4.11E-19	2.36E-18
AC015982.2	0.464077292	0.946197579	1.027776363	1.55E-10	3.16E-10
AC007099.1	0.013202506	0.612755784	5.53642849	1.02E-07	1.57E-07
AL031673.1	0.495193713	1.843383067	1.896291001	2.06E-19	1.25E-18
SNHG32	9.754471395	36.20809294	1.892176594	3.75E-25	1.33E-23
RUSC1-AS1	0.286732541	1.654774681	2.528857237	1.14E-24	3.27E-23
AC008771.1	1.045183744	2.529413913	1.275046548	5.02E-19	2.82E-18
CRIM1-DT	0.851885881	3.148073329	1.885737063	0.000111587	0.00014303
LINC01004	0.201822868	0.842962097	2.062378109	2.09E-22	2.68E-21
DLG5-AS1	0.179581964	1.38573567	2.947937721	1.56E-23	2.94E-22
MUC20-OT1	0.327767438	0.844240262	1.364981095	5.80E-18	2.62E-17
AP000894.4	0.374368665	1.259622347	1.750459666	3.26E-13	8.46E-13
ZEB1-AS1	0.274887624	0.805408081	1.550877991	3.63E-22	4.44E-21
ZNF503-AS2	0.411786638	1.040383029	1.337145847	1.67E-17	7.11E-17
AL445524.1	1.830088172	12.66057767	2.79035817	8.48E-26	4.52E-24
AL354836.1	0.487700152	1.496240045	1.617275321	2.75E-15	8.89E-15
AP002748.4	0.545956334	1.294132466	1.245127826	2.48E-14	7.21E-14
lnc-CCNY-1	0.237875227	0.826470642	1.79675854	5.49E-19	3.01E-18
ZNF793-AS1	0.100200451	0.571154517	2.510992099	0.00059752	0.000727162
FAM99B	3.774539922	1.807265988	-1.062491952	1.03E-14	3.13E-14
AC009022.1	0.334901519	0.709270635	1.082599299	1.28E-10	2.63E-10
AL162413.1	0.011029778	4.245629257	8.588430832	3.05E-09	5.40E-09
AL035071.1	0.803632974	1.998842698	1.314556275	6.45E-15	2.01E-14
AC012146.1	0.325398415	1.504259026	2.208773882	3.99E-20	2.81E-19
AC009275.1	0.087307501	0.733148381	3.069927708	1.02E-08	1.72E-08
AP001372.2	0.554062806	1.199577983	1.114405521	1.22E-15	4.10E-15
AL358472.2	0.389858988	0.892931563	1.195597213	4.37E-16	1.61E-15
AC139530.1	0.504626935	1.532106419	1.60222739	9.25E-21	7.34E-20
AC022424.1	0.008662141	0.788275526	6.50783245	1.47E-07	2.24E-07
AL354892.2	1.056558067	2.284709004	1.112638367	8.24E-08	1.28E-07
KCNMB2-AS	0.017109487	0.749220675	5.452522275	1.26E-14	3.79E-14
MID1IP1-AS1	0.322095583	1.010145552	1.649002403	2.55E-09	4.56E-09
NOP14-AS1	0.508030851	1.086812758	1.09711539	4.54E-21	4.40E-20
C2-AS1	0.245821627	0.62803762	1.35323913	6.06E-08	9.57E-08

AC068888.1	0.742697028	1.509608604	1.02332884	1.96E-10	3.92E-10
BX322562.1	0.793129214	2.019644165	1.348473302	0.016541561	0.018696976
SNHG19	10.30830584	26.11049965	1.340822818	1.57E-06	2.28E-06
AL121899.1	0.318121081	2.390599329	2.909724465	1.11E-19	7.07E-19
AC007773.1	0.137650204	0.707799145	2.362333274	4.93E-18	2.26E-17
LINC02298	0.129878478	0.703708669	2.437815901	8.98E-05	0.000116145
PARD3-AS1	0.210160208	0.590742124	1.491038959	1.97E-08	3.24E-08
AC145207.5	0.195688849	0.684147936	1.805746768	4.30E-19	2.45E-18
AC087741.1	0.184839988	0.716305268	1.954297649	6.13E-19	3.34E-18
AC024075.1	0.17466264	0.54990291	1.654605872	5.20E-10	1.00E-09
AC022144.1	0.523767162	1.218032614	1.217555247	0.000648466	0.000787876
AL360181.2	0.334574234	0.846638556	1.339419844	5.23E-10	1.00E-09
AC027644.3	1.313398687	2.823768982	1.104317145	8.82E-08	1.36E-07
LINC00513	0.157960865	0.647674503	2.035701779	5.61E-07	8.30E-07
BAIAP2-DT	1.719264129	4.877977405	1.504491874	8.92E-19	4.59E-18
SNHG10	0.455101749	1.518934279	1.738798413	1.56E-23	2.94E-22
AC004687.1	0.196520938	0.638454567	1.69990093	1.53E-06	2.23E-06
STPG3-AS1	0.13922199	0.774310088	2.475524334	2.17E-14	6.33E-14
SNHG29	18.16455582	37.66017102	1.051913464	5.89E-08	9.37E-08
AC023043.4	0.252394816	0.890744932	1.819330092	6.65E-13	1.68E-12
MAP3K2-DT	0.419094722	1.008296499	1.266571682	1.30E-09	2.42E-09
AC027307.2	2.892197113	8.621630507	1.575794855	4.19E-24	9.77E-23
KMT2E-AS1	2.387686128	5.900016388	1.305105761	1.52E-13	4.04E-13
BX537318.1	0.477849217	1.42375841	1.575077005	6.92E-21	6.00E-20
AC020915.3	0.384037725	1.193137149	1.635439946	1.47E-22	1.92E-21
AL356234.2	0.053979861	0.826058104	3.935750086	7.62E-14	2.06E-13
AL392172.1	1.848659659	5.774382711	1.643187082	2.30E-21	2.42E-20
CCDC18-AS1	0.248727066	1.024183795	2.041839226	6.26E-23	9.34E-22
AC138696.2	0.586640336	2.541743588	2.115270322	2.28E-20	1.65E-19
MINCR	0.769633169	3.339484281	2.117382441	5.23E-21	4.70E-20
RPARP-AS1	0.721190597	1.489844335	1.046709108	4.39E-14	1.23E-13
AC103691.1	0.443404798	0.929335187	1.067574651	8.70E-08	1.35E-07
LINC00685	0.125698582	0.870312455	2.791565069	3.60E-23	5.72E-22
AL139287.1	0.856954718	2.656585584	1.632282313	2.35E-21	2.44E-20
ALDH1L1-AS	0.319395703	0.689546531	1.110303003	0.003118008	0.003640116
AC040977.1	1.058995171	3.028163359	1.515747025	3.00E-18	1.42E-17
CYTOR	0.722748445	4.130731915	2.514831927	8.43E-21	6.98E-20
AC115618.2	2.291424543	4.702419198	1.037158376	5.94E-11	1.29E-10
MELTF-AS1	0.112246069	0.96102527	3.097909452	7.08E-21	6.07E-20
SNHG1	1.243861057	6.041753795	2.280142053	4.47E-26	3.03E-24
AC104113.1	0.486284395	1.070232158	1.138051584	9.24E-12	2.08E-11
GPRC5D-AS1	0.401210526	0.934750913	1.220222518	1.66E-19	1.03E-18
LINC01857	0.259607692	0.534094749	1.040762573	0.000205374	0.000259236
AC010331.1	0.119087887	0.606362044	2.348152775	7.63E-22	8.76E-21

AC005332.4	0.232729234	0.563754692	1.276415089	1.78E-11	3.96E-11
AC000123.1	0.433409077	0.959739735	1.146913854	1.65E-12	4.01E-12
AL035461.3	0.370122689	2.059543869	2.476249373	6.52E-20	4.38E-19
AL022328.2	0.734639296	2.099015186	1.514604631	6.21E-17	2.48E-16
CAMTA1-DT	0.242565786	0.593917673	1.291886893	1.16E-07	1.78E-07
AL118516.1	1.739652845	3.558272014	1.032377363	2.12E-10	4.22E-10
AC132872.1	0.521036339	1.942457047	1.898426798	1.87E-23	3.41E-22
SREBF2-AS1	0.190006441	0.929223494	2.289977308	2.58E-26	2.14E-24
AC147067.1	0.332910613	0.811312853	1.285123482	1.07E-05	1.47E-05
AC091271.1	0.670871395	1.479531876	1.141032644	2.30E-08	3.75E-08
AC020978.4	4.697383269	2.171596055	-1.113101543	1.64E-15	5.36E-15
AC124016.3	0.256977606	0.622836289	1.27721036	6.96E-07	1.03E-06
FAM111A-DT	0.27312099	0.698282152	1.354269903	7.48E-17	2.97E-16
AL022328.1	0.317448984	0.790558033	1.31634661	1.97E-11	4.35E-11
TMEM147-AS	0.183057555	0.685394666	1.904637648	3.38E-22	4.20E-21
AC012313.1	0.60987427	1.304764514	1.097205693	9.70E-17	3.77E-16
AP000424.2	0.121326288	0.591554043	2.285617805	0.001875354	0.002206647
NCK1-DT	0.636228195	1.416333768	1.154545072	2.25E-19	1.36E-18
AL158071.3	0.203915659	0.619442837	1.602998591	3.73E-10	7.28E-10
AL928654.2	0.307820723	0.916629928	1.57424903	9.11E-12	2.07E-11
AC019117.2	0.2460559	0.899712611	1.870478137	0.000428647	0.000526806
AC068473.5	0.41929234	1.157337812	1.464781652	1.80E-18	8.89E-18
SNHG7	1.412275112	6.609582744	2.226538045	1.87E-24	4.98E-23
CR936218.2	0.312460611	0.861486557	1.463153953	0.001408095	0.001678017
LINC00957	0.319172783	0.731721487	1.19695699	3.95E-08	6.36E-08
CD2BP2-DT	1.001605303	2.272651853	1.182062589	7.14E-12	1.63E-11
LINC01963	0.28627718	0.853750498	1.576401839	9.60E-15	2.94E-14
AC015813.1	0.209154727	0.796465338	1.92904097	6.08E-17	2.44E-16
AL023803.1	0.581269117	1.525726291	1.392218007	2.43E-05	3.27E-05
AC006504.7	0.20183604	0.729382202	1.853491191	7.10E-16	2.53E-15
APTR	1.110511786	2.650321923	1.254942903	1.32E-22	1.75E-21
LINC00106	0.374020238	1.643099847	2.13523191	1.20E-15	4.04E-15
SNHG9	3.260920702	9.528414378	1.546956797	1.33E-10	2.73E-10
TAF1A-AS1	0.747174453	1.499026046	1.004508417	1.23E-09	2.30E-09
AC008267.5	2.828380459	6.126456751	1.115076732	6.67E-11	1.43E-10
AC009065.9	0.518518145	1.219768723	1.234141249	5.97E-15	1.87E-14
AC008610.1	0.197255472	1.030346752	2.384992713	9.02E-19	4.61E-18
AL161668.4	6.377864747	2.548529989	-1.323408174	4.74E-16	1.73E-15
AC026462.3	1.320374487	3.736760486	1.500840927	5.91E-05	7.74E-05
EIF3J-DT	0.46914957	1.015647739	1.114280267	7.32E-16	2.57E-15
PURPL	0.08166707	0.662927988	3.02102579	2.02E-08	3.33E-08
LINC01980	0.0056925	1.764768061	8.276200571	2.59E-08	4.21E-08
MIR210HG	0.439850653	1.419384255	1.690179548	0.000416062	0.000512182
OTUD6B-AS1	1.621410083	3.280372408	1.016610588	3.27E-18	1.53E-17

AC016394.2	0.13096983	0.71975928	2.458279967	1.09E-25	5.06E-24
AL031058.1	2.064091804	4.936446885	1.257965866	3.36E-05	4.47E-05
AC009237.14	1.338442687	3.017227212	1.172667979	1.64E-15	5.36E-15
AC010761.1	0.219843321	0.749768758	1.769970009	8.80E-18	3.91E-17
AP001505.1	1.278711791	3.855313685	1.592157117	1.77E-12	4.28E-12
AC132872.3	0.248567626	0.632864137	1.348257405	1.93E-09	3.48E-09
AC022150.2	0.203157308	0.54793805	1.431415532	0.000316916	0.00039469
AC083880.1	0.202428576	0.676264462	1.740174576	1.55E-14	4.62E-14
AC016888.1	2.416702281	6.058207816	1.325851313	2.70E-12	6.46E-12
LINC01003	0.851390262	2.042197408	1.262229837	6.21E-15	1.94E-14
AL359643.3	0.336933172	0.836039018	1.311107801	3.18E-14	9.09E-14
AC079305.2	0.147634218	0.583040348	1.981568585	1.24E-07	1.89E-07
MAPKAPK5-/	1.350741717	3.967566979	1.554502744	8.83E-27	9.41E-25
AC004540.2	1.749295683	0.567111353	-1.625070225	8.08E-20	5.33E-19
AP000593.3	0.023459695	1.496655086	5.995413725	1.27E-10	2.62E-10
AP000254.2	1.254763166	2.521725257	1.006996018	6.47E-13	1.64E-12
LINC02806	0.001134756	0.717886145	9.305229585	1.17E-15	3.98E-15
AC060780.1	0.228524645	0.809819415	1.825250474	2.87E-12	6.85E-12
SNHG14	0.211368991	0.637834183	1.593417677	0.006128952	0.007066767
AL589765.4	0.366775304	1.161204531	1.662653701	3.10E-14	8.94E-14
AC087482.1	6.581079093	16.04875691	1.286065489	1.73E-09	3.12E-09
AC027796.4	0.106293957	0.583389064	2.456398756	1.25E-17	5.46E-17
LINC00221	0.00271213	1.104430429	8.669660654	1.59E-07	2.40E-07
AL391427.1	0.333895911	1.418698148	2.087097334	1.39E-11	3.12E-11
LINC00665	0.119604438	1.405468455	3.55470824	1.62E-10	3.29E-10
PTOV1-AS2	0.453242904	1.556879788	1.780301217	2.66E-18	1.27E-17
SNHG11	1.670991329	5.161074219	1.626967131	3.39E-23	5.62E-22
PPP1R14B-AS	0.292719134	1.214101557	2.052300145	1.39E-14	4.15E-14
AL121832.2	0.77911471	2.727053058	1.807435111	1.49E-19	9.40E-19
AC005920.4	0.458070454	1.228888707	1.423712851	0.00037545	0.000464487
AC010542.6	0.236220316	0.873335255	1.88640253	1.17E-18	5.92E-18
HEIH	5.341753754	12.86642167	1.268225499	2.07E-24	5.32E-23
AC107375.1	0.162585019	0.732006964	2.170663042	9.91E-26	4.93E-24
AL391056.1	0.0226113	0.671633212	4.892557719	1.40E-07	2.13E-07
FOXD2-AS1	0.225471947	1.579584669	2.808525422	5.05E-21	4.59E-20
LINC01089	0.248189245	1.233952733	2.313774631	3.82E-26	2.85E-24
AL355802.3	0.122381606	0.583838247	2.254181989	5.07E-20	3.54E-19
LINC01436	0.088615483	1.690845234	4.254042011	5.70E-06	7.91E-06
MAGI2-AS3	1.564101827	0.758725061	-1.043685342	1.16E-13	3.10E-13
AC092171.3	0.222339359	0.849430493	1.933732542	4.57E-18	2.12E-17
AL591895.1	2.803405997	10.15936987	1.857558323	3.09E-21	3.11E-20
AC074117.1	0.472669371	1.294549403	1.453546735	4.76E-21	4.50E-20
LINC02163	0.001209671	1.021285824	9.721556245	1.64E-14	4.85E-14
PVT1	0.118649012	0.929566969	2.96985872	2.90E-20	2.08E-19

AC125257.1	1.260417867	2.688781991	1.093050675	1.38E-21	1.47E-20
AC008764.2	0.420982015	1.184436774	1.492370684	8.15E-11	1.71E-10
RNASEH1-AS	1.30650646	2.769461051	1.083890991	9.79E-16	3.41E-15
NRSN2-AS1	0.361432325	0.952883061	1.398573637	4.25E-12	9.92E-12
AC009283.1	1.814617584	4.569689236	1.332430513	7.56E-19	4.00E-18
CHKB-DT	0.521489299	1.286360627	1.302585601	2.63E-16	9.91E-16
PANK2-AS1	0.235169892	0.654007651	1.475604144	4.43E-13	1.13E-12
LINC01484	0.203282517	0.553471357	1.445022515	1.46E-09	2.66E-09
GIHCG	0.389109542	1.925016018	2.306622187	3.99E-24	9.59E-23
SNHG17	1.428560482	3.703285708	1.37424374	3.19E-16	1.20E-15
ST8SIA6-AS1	0.025064855	2.103294092	6.390840914	1.88E-10	3.77E-10
AC104825.1	0.409677311	0.97451432	1.250195392	1.07E-10	2.23E-10
LINC00294	0.86222429	1.87437296	1.120272936	2.84E-18	1.35E-17
AC006449.7	0.636534607	1.282470752	1.010615067	1.33E-09	2.47E-09
AC006213.4	0.253244531	0.839264129	1.728593804	3.29E-10	6.49E-10
AC084824.6	0.330473975	0.850634356	1.36400246	1.71E-15	5.55E-15
AC015849.3	0.184326235	0.565450406	1.617139076	7.65E-10	1.46E-09
AC111000.4	0.122344582	1.127003489	3.203469859	1.78E-17	7.53E-17
RAD51-AS1	0.343280849	1.056517294	1.621855104	7.47E-20	4.97E-19
AC024060.2	0.869129721	2.733276196	1.652987824	8.19E-23	1.17E-21
POLR2J4	0.618527154	1.265251089	1.032514878	1.01E-16	3.91E-16
MHENCR	1.642990436	4.408031555	1.423810469	4.83E-17	1.96E-16
LINC01474	0.368580722	2.549065218	2.789915765	0.004541402	0.005260692
AL031186.1	0.123074122	0.595767219	2.275221297	4.87E-21	4.54E-20
AC005229.4	0.660627422	1.68163126	1.347952631	1.86E-19	1.14E-18
AP000759.1	0.605085004	1.537360252	1.345245539	3.18E-14	9.09E-14
AC074212.1	0.153294065	0.633311106	2.046612539	7.80E-23	1.14E-21
BX284668.5	3.892016712	8.167326972	1.069346081	1.26E-08	2.12E-08
AL391244.2	0.351645955	1.049788356	1.577902974	1.31E-20	9.87E-20
AL355488.1	0.159005364	0.990814249	2.639539177	9.07E-25	2.71E-23
AC008915.3	0.921069686	1.967003793	1.094617523	1.56E-15	5.19E-15
AP003119.2	0.523414296	1.745231194	1.73739293	2.72E-08	4.41E-08
AL160006.1	0.215474404	0.817242232	1.923247254	7.63E-22	8.76E-21
GLIS2-AS1	0.474825944	1.679786897	1.82280755	4.05E-06	5.66E-06
AL844908.2	0.221688181	0.550803821	1.313006711	1.09E-07	1.68E-07
SBF2-AS1	0.233661797	0.755555693	1.693116224	3.38E-22	4.20E-21
MIR4458HG	0.666210078	1.560526303	1.227983592	1.95E-09	3.50E-09
AL118558.3	0.591829213	1.336730367	1.175455671	1.64E-12	3.99E-12
ASH1L-AS1	0.294662667	0.774383771	1.393984432	5.26E-18	2.39E-17
AC012645.1	0.260345118	0.666269675	1.355680876	2.71E-13	7.06E-13
AL021707.6	0.447573723	1.213483787	1.438957589	5.47E-12	1.26E-11
AC092535.5	1.036944631	6.471465378	2.641753567	7.00E-19	3.73E-18
AC009686.2	0.171282346	0.670774825	1.969452082	7.12E-11	1.52E-10
LINC01176	0.436530348	1.21068236	1.471666543	1.29E-15	4.33E-15

AP003352.1	0.373764655	1.541082309	2.043741865	2.65E-23	4.49E-22
C2CD4D-AS1	1.028418254	4.638335288	2.173179988	5.25E-19	2.92E-18
AC242426.2	0.268882146	0.904868493	1.750734172	5.19E-20	3.59E-19
AC099508.2	1.932978829	0.547204105	-1.820674878	4.34E-19	2.45E-18
AC026740.1	0.138208117	1.355019526	3.293399393	2.29E-19	1.37E-18
AC022784.1	0.246774421	1.013012321	2.037386952	5.16E-07	7.69E-07
LINC01006	0.91429656	2.080160882	1.185961015	1.01E-15	3.50E-15
SNHG3	0.633348786	3.314609166	2.38776665	3.52E-23	5.70E-22
AC004918.5	0.394433225	1.168577473	1.566900396	4.77E-18	2.20E-17
AL355574.1	0.219372773	0.917609601	2.064496005	6.62E-19	3.55E-18
SCAMP1-AS1	1.018731232	2.342763725	1.20143798	9.69E-20	6.29E-19
AL512598.1	0.115340948	0.653987553	2.503358392	8.57E-09	1.46E-08
DLGAP1-AS1	1.271828122	3.068001657	1.270395548	2.09E-10	4.17E-10
MIR4435-2HG	0.372212169	2.014476078	2.436207543	9.95E-23	1.40E-21
AP002360.2	1.214584404	4.067104015	1.743539138	8.08E-19	4.24E-18
AC013275.1	0.098836092	0.983149199	3.314300494	1.05E-07	1.62E-07
LINC00847	2.098642201	4.264080715	1.022778526	3.39E-16	1.27E-15
VPS9D1-AS1	0.293665732	0.823904833	1.488302779	1.16E-06	1.70E-06
LINC02041	0.080913218	1.092878465	3.755613765	0.002728863	0.003195811
PRRT3-AS1	0.442701121	1.74838108	1.981614738	2.87E-10	5.68E-10
AC010883.1	0.422618433	1.250779865	1.565400302	6.47E-13	1.64E-12
PSORS1C3	0.144937659	0.911937382	2.653502263	1.35E-09	2.49E-09
AC106820.3	0.182073832	0.936767927	2.363168094	6.44E-19	3.48E-18
AC136475.2	0.532915238	1.350710813	1.341740835	7.27E-13	1.83E-12
AC026471.4	0.700185067	1.612986001	1.203925719	3.73E-14	1.06E-13
AC016773.2	0.060701386	0.57856949	3.252688877	3.47E-25	1.33E-23
LINC02604	0.686023799	1.567988833	1.192584754	3.43E-13	8.83E-13
AC007038.1	0.177518717	0.604213336	1.767086879	3.11E-19	1.84E-18
AC010326.3	1.238888332	2.959211182	1.256166502	4.83E-17	1.96E-16
AC243964.3	0.76591208	1.811366639	1.241827894	1.96E-06	2.82E-06
ARRDC1-AS1	1.527504804	3.569037691	1.22435822	8.45E-24	1.80E-22
MMP25-AS1	0.293516831	0.729783568	1.314025434	3.40E-13	8.78E-13
GAS5	6.02765298	33.72981137	2.484355984	3.75E-25	1.33E-23
LINC01186	0.07648313	0.778008193	3.346571879	1.61E-14	4.78E-14
AC132192.2	0.117528538	0.973848204	3.050685804	4.78E-28	3.46E-25
AC007405.4	0.903435708	2.434416381	1.430082104	8.72E-15	2.69E-14
HCP5	3.080273133	8.096910048	1.394313167	1.13E-09	2.13E-09
AP002761.4	0.174445436	0.759199839	2.121703835	7.92E-12	1.80E-11
AC009779.3	1.793327944	3.783867971	1.077222414	1.42E-16	5.44E-16
IDH1-AS1	0.466802571	1.204681077	1.36776685	1.70E-09	3.09E-09
AL162595.1	0.249470838	0.770419963	1.626773904	1.36E-17	5.88E-17
LINC00853	0.287481801	2.16617787	2.913609178	1.23E-21	1.33E-20
LENG8-AS1	0.190327248	0.882615372	2.213302754	1.87E-24	4.98E-23
AC008443.5	0.643931135	1.837000871	1.512373998	4.13E-10	8.03E-10

AC005261.3	1.477344925	2.973787727	1.009294969	9.39E-18	4.12E-17
AC022306.2	0.302516035	1.223073082	2.01542709	1.27E-22	1.75E-21
AC142472.1	0.21840088	0.663329078	1.602746102	5.37E-19	2.97E-18
SNHG21	0.193549448	0.642201266	1.730323318	6.92E-21	6.00E-20
AC011445.2	0.303189394	2.805597689	3.210016957	8.52E-21	6.98E-20
AC005840.4	0.281507481	0.568706594	1.01451127	1.34E-08	2.24E-08
NEAT1	5.481794673	12.64570131	1.205926853	9.87E-09	1.68E-08
AC016394.3	0.189737859	0.830290432	2.129608503	8.24E-21	6.90E-20
AC012313.9	0.864041361	1.757568206	1.024408398	9.24E-07	1.36E-06
AC103706.1	0.254451288	1.342625954	2.399596039	7.37E-21	6.25E-20
ARHGAP27P1	0.177378822	0.58458452	1.720577858	8.63E-21	7.00E-20
AC068580.3	0.311358987	1.017235458	1.708002832	3.09E-14	8.93E-14
AL590666.2	0.176694271	2.469023349	3.804613307	2.09E-16	7.92E-16
PRR34-AS1	0.935418166	3.428075084	1.873715357	2.02E-13	5.30E-13
ST7-AS1	0.206385609	0.545946436	1.403417034	9.95E-11	2.08E-10
TBX2-AS1	0.171173754	0.777199041	2.182822608	9.24E-12	2.08E-11
AC005332.5	0.35977232	1.790951899	2.315570493	1.06E-26	9.92E-25
AL359504.1	0.175786082	0.544794729	1.631891892	6.39E-14	1.75E-13
LINC02035	0.291287034	0.61888106	1.087220688	1.71E-10	3.45E-10
LINC02027	5.577540796	1.867270798	-1.578697993	1.67E-19	1.03E-18
AP003486.1	0.341557722	0.827402843	1.276460506	1.61E-15	5.30E-15
AC012467.2	0.571004256	1.269548577	1.152742195	5.50E-20	3.76E-19
AC006026.3	0.270700234	0.648451532	1.260302612	0.001284124	0.001532731
AC010973.2	0.110304907	0.626642246	2.50614507	7.06E-26	4.05E-24
ITGB1-DT	0.566652551	1.563889238	1.464602031	0.001060006	0.001271325
AL365181.3	0.226268093	4.241738086	4.22855048	7.58E-18	3.39E-17
AC011700.1	0.233290374	0.548132885	1.232398911	3.56E-06	4.99E-06
AC008735.2	0.370896114	1.546772708	2.060174156	6.96E-16	2.49E-15
AC068506.1	0.120930405	0.753633091	2.639685291	0.000147972	0.000187733
AC011477.2	0.542014339	1.396629806	1.365546743	2.86E-09	5.10E-09
AL121845.4	0.564475392	2.073724974	1.877241977	2.26E-06	3.22E-06
U91328.1	0.472911956	1.228561724	1.377326819	2.13E-14	6.26E-14
AC005332.3	1.737847658	4.635553191	1.415439896	2.31E-18	1.11E-17
AL050341.2	0.901120996	2.179594979	1.274267334	7.24E-16	2.56E-15
AC005261.1	1.635384881	3.932949574	1.265981481	2.65E-23	4.49E-22
AC139100.2	0.144008588	0.980063702	2.766720671	6.93E-22	8.34E-21
AC012615.1	0.456284337	1.737742556	1.929209329	1.10E-23	2.28E-22
ASB16-AS1	0.835191588	2.624522677	1.651875977	2.86E-25	1.19E-23
AL109615.3	0.045130976	0.733589182	4.02278249	1.82E-11	4.03E-11
LINC01549	0.128304699	2.128593723	4.052254701	0.000559799	0.000683486
AC087741.2	0.352440521	1.302650383	1.885998225	1.72E-13	4.56E-13
ZNF687-AS1	0.677051958	1.868192289	1.464304499	4.37E-15	1.39E-14
AC007566.1	0.222305364	0.549458697	1.305468276	4.23E-09	7.38E-09
Z98257.1	0.170202288	0.806714971	2.244808595	1.08E-08	1.82E-08

AJ009632.2	0.319345776	1.330368067	2.058634171	0.020946623	0.023604503
CEBPA-DT	1.439060475	4.884097309	1.762964724	3.55E-15	1.14E-14
AC026369.2	0.198161928	0.548709301	1.469362222	1.89E-13	4.99E-13
AL162411.1	1.669100818	3.850118343	1.205831691	8.23E-09	1.41E-08
AC008608.2	0.97035516	2.1297413	1.134093405	1.73E-09	3.12E-09
ARHGAP5-AS	0.443274539	0.929613641	1.068430738	8.25E-11	1.73E-10
AC010719.1	0.187139395	2.053147927	3.455652374	5.69E-26	3.54E-24
AL441992.2	1.048315024	3.273099831	1.642585287	1.85E-20	1.37E-19
LINC00205	0.28934551	1.405556398	2.28027618	1.03E-21	1.14E-20
TRIM52-AS1	2.198440573	4.953459416	1.171955897	1.09E-15	3.72E-15
AC064807.4	0.280908194	0.688471886	1.293299032	6.04E-08	9.56E-08
AC078993.1	0.026048775	0.834589155	5.001778747	3.55E-10	6.96E-10
AP000240.1	0.213292759	0.639437556	1.583968489	1.17E-09	2.19E-09
SH3BP5-AS1	0.32205344	0.662067007	1.039677136	1.34E-09	2.48E-09
AC023509.3	1.118650355	2.483156223	1.150415851	1.64E-10	3.33E-10
AC002456.1	0.529780271	1.384336111	1.385728242	8.84E-05	0.000114477
AC127024.5	0.187298956	0.878004363	2.228885251	9.47E-21	7.44E-20
AC015912.3	0.368841705	1.482855777	2.007304591	7.30E-11	1.55E-10
AC099850.4	0.183166256	2.081553606	3.506435065	3.81E-18	1.78E-17
AC244090.1	3.362676672	9.028119138	1.42481539	1.98E-17	8.19E-17
AC084018.1	0.231807989	1.102951651	2.250367358	9.36E-17	3.66E-16
LINC01093	30.82676897	4.466403791	-2.786998094	2.29E-25	1.01E-23
AC010531.6	0.378411652	0.970834631	1.359269061	1.94E-06	2.79E-06
ZNNT1	0.370407384	1.615050871	2.124394846	1.39E-20	1.04E-19
AC110285.2	0.228375502	1.539481976	2.75296517	3.56E-19	2.09E-18
AL365330.1	0.376062086	0.925295021	1.298942564	1.51E-11	3.39E-11
ELFN1-AS1	0.045659788	0.910601674	4.317824032	0.005868812	0.006787804
AC008736.1	0.374566423	1.090305671	1.541439174	2.29E-12	5.50E-12
AL031985.3	0.218938548	0.723300283	1.72406873	8.18E-20	5.35E-19
AC109322.1	0.245663439	1.545493891	2.653312885	1.87E-27	3.49E-25
AL135999.1	0.165958864	0.647764127	1.964642884	1.89E-17	7.89E-17
AL365203.2	0.682079331	2.228083578	1.707791901	3.48E-15	1.12E-14
LINC00997	0.581848149	1.178156961	1.017817164	8.01E-16	2.81E-15
AC010280.2	3.545020747	0.881214539	-2.008228867	3.36E-21	3.30E-20
AC084125.4	0.179534961	0.671596199	1.903329255	1.08E-14	3.27E-14
AC253536.6	0.418572703	1.685761684	2.009850462	3.37E-09	5.93E-09
AC007996.1	0.225624513	0.713521126	1.66103233	1.58E-15	5.22E-15
LINC02241	0.000425954	0.888620909	11.02665541	1.28E-12	3.15E-12
U62317.2	1.545934228	3.76831011	1.285438755	5.74E-16	2.08E-15
HAGLR	0.011622331	1.507578988	7.019190308	1.39E-27	3.46E-25
CTBP1-DT	0.738000615	1.79760264	1.284380225	1.00E-21	1.13E-20
AC005696.1	0.365970408	0.830937088	1.183012253	1.47E-08	2.44E-08
MAFG-DT	0.119232737	1.476454438	3.63028453	7.98E-25	2.48E-23
AC015871.3	0.359205171	0.80854122	1.170513208	5.86E-13	1.50E-12

AC023090.1	0.007746404	0.626512415	6.337672465	7.54E-09	1.30E-08
AC007541.1	0.399440472	1.043334092	1.385148783	1.04E-15	3.57E-15
AC064836.2	0.48865458	1.086648122	1.152997924	7.08E-10	1.35E-09
AC007406.2	0.160209815	1.189948631	2.892864855	1.98E-06	2.83E-06
AC095057.3	0.171051357	0.559145661	1.708794616	2.95E-12	7.01E-12
LUCAT1	0.021122813	0.57882412	4.776251183	1.12E-08	1.89E-08
ZFAS1	6.459190071	19.5377496	1.596839125	1.38E-17	5.91E-17
SNHG30	1.354942417	3.94449477	1.541608984	8.44E-19	4.37E-18
LINC01703	0.124766739	1.046642993	3.068464132	2.05E-20	1.50E-19
AC004816.1	0.145397766	0.996639123	2.777066105	1.32E-22	1.75E-21
AC004148.1	0.262707804	0.790841278	1.589929119	1.07E-15	3.67E-15
WNT5A-AS1	0.191036281	0.608340758	1.671033007	0.003879301	0.004507724
AC024075.2	0.804419872	2.408967513	1.582394311	4.93E-21	4.54E-20
AL117379.1	0.126153369	0.619513827	2.295957745	1.10E-21	1.21E-20
AC084824.5	0.428670073	1.024414475	1.256859938	1.66E-13	4.42E-13
AC002398.1	0.309953994	0.89412751	1.528426491	4.96E-15	1.57E-14
AC048341.2	0.215024899	1.087920841	2.338997956	3.03E-21	3.10E-20
AL133353.1	0.382270755	1.009005152	1.400266804	1.99E-09	3.57E-09
AC016747.1	1.090885513	2.809531913	1.364830087	8.60E-27	9.41E-25
AC104958.2	3.574542894	12.10274127	1.759503185	3.24E-14	9.23E-14
YTHDF3-AS1	0.868331048	2.105716105	1.277993869	1.34E-10	2.74E-10
AL390728.6	0.992260074	3.99979806	2.011136954	1.78E-18	8.85E-18
DSCR8	0.00244764	1.188884955	8.924001807	8.02E-09	1.38E-08
LINC00623	0.739845703	1.692710584	1.194038997	2.17E-11	4.77E-11
LINC01770	0.756309157	1.989616771	1.395442583	2.32E-07	3.47E-07
AC090587.1	0.486698677	1.009292034	1.052242913	4.96E-08	7.92E-08
BACE1-AS	0.560946135	2.149493231	1.93806242	5.04E-25	1.71E-23
AP003119.1	0.195245302	0.959530531	2.297040877	3.04E-09	5.39E-09
SNHG25	0.651616432	5.018618531	2.945195399	1.99E-23	3.54E-22
AC120053.1	0.550379964	1.628418854	1.564971974	6.09E-20	4.13E-19
LINC00342	0.389268718	1.131617874	1.539548553	2.17E-14	6.33E-14
AL603839.3	0.272784641	0.73406321	1.428141882	4.54E-09	7.88E-09
AL359921.2	0.474473608	1.734023842	1.869723987	1.10E-20	8.56E-20
PRANCR	0.41516073	1.046368482	1.333649099	9.09E-18	4.01E-17
LINC02062	0.207210491	0.548325263	1.403934893	1.58E-11	3.53E-11
ANKRD10-IT	0.853367549	2.269042412	1.410844419	1.66E-07	2.51E-07
AC011477.1	0.378294556	0.986701954	1.383104352	2.25E-07	3.37E-07
AC084036.1	1.12804949	3.565437322	1.660248679	4.51E-16	1.66E-15
AC083843.3	0.297522336	0.754352715	1.342241268	1.38E-08	2.29E-08
AFDN-DT	0.31698972	0.69187532	1.126076025	1.38E-09	2.54E-09
AC131009.3	0.451700065	1.221894441	1.435682632	1.75E-12	4.23E-12
LMNTD2-AS1	0.195996941	0.824573498	2.072816959	7.63E-14	2.06E-13
AC090015.1	0.001594639	0.891997267	9.127665984	6.85E-11	1.47E-10
AL360181.1	1.31039534	2.764977981	1.07726586	0.027017364	0.030037189

SNHG20	0.361795401	1.288704254	1.832675243	3.89E-24	9.59E-23
AC092171.5	0.415698096	1.573044823	1.919951736	1.35E-17	5.85E-17
LINC01270	0.629098944	1.424774549	1.179374805	2.59E-13	6.77E-13
AC112206.2	2.48397517	1.192771271	-1.058333338	3.70E-12	8.68E-12
AL121944.2	1.261878189	2.52790702	1.002370748	6.35E-11	1.37E-10
LINC01836	0.229212304	0.878819225	1.938881939	0.004285153	0.004971577
AC055822.1	0.221619813	0.704283912	1.66807026	4.86E-14	1.35E-13
AC009005.1	0.189460554	1.413915171	2.899726156	3.69E-20	2.62E-19
TMEM161B-A	0.402836291	1.107876099	1.459530978	1.25E-18	6.28E-18
AC010969.2	0.527831155	1.193936672	1.177577904	1.85E-17	7.82E-17
LINC01503	0.55685278	1.431528286	1.362188311	0.002720992	0.003191604
AL132989.1	0.29530129	0.661630484	1.163838049	1.61E-07	2.43E-07
LINC01124	0.949324823	3.414408658	1.846662027	2.77E-05	3.70E-05
AC092171.2	0.320882438	3.082692473	3.264074239	1.15E-27	3.46E-25
PXN-AS1	0.720148228	1.894677722	1.39558668	4.65E-21	4.45E-20
SNHG6	11.70091059	45.53758456	1.960436961	3.16E-21	3.14E-20
AC007448.4	0.264069376	0.772097766	1.547866538	1.03E-09	1.95E-09
ELF3-AS1	0.433825204	2.177377172	2.32740556	1.04E-19	6.67E-19
LINC01138	0.226352932	1.055284413	2.220985978	5.96E-23	9.07E-22
AC010735.2	0.39527788	1.056607408	1.418500302	1.02E-09	1.92E-09
COA6-AS1	1.036828394	3.322924388	1.680276333	7.34E-18	3.30E-17
AC021078.1	0.380295507	0.829083598	1.124396685	9.11E-14	2.45E-13
NIFK-AS1	0.570199117	1.264887373	1.149471222	3.89E-19	2.27E-18
LINC01287	0.004107994	2.936954242	9.48167102	3.81E-10	7.43E-10
AC026401.3	0.913816837	3.270510914	1.839539099	2.24E-18	1.08E-17
TYMSOS	0.779702209	2.927928488	1.90888519	3.63E-14	1.03E-13
PTOV1-AS1	0.473181926	1.231985274	1.380518137	4.84E-23	7.52E-22
TMEM44-AS1	2.565958159	5.85702141	1.190669522	1.52E-12	3.74E-12
UBR5-AS1	0.675681847	1.838510237	1.444121209	1.57E-23	2.94E-22
PRKAR1B-AS	0.312737134	0.842777698	1.430201606	1.88E-08	3.12E-08
MALAT1	2.157594368	6.152177036	1.511673358	1.04E-15	3.57E-15
SLC25A25-AS	0.693351964	1.842662328	1.410131924	8.60E-10	1.63E-09
LINC01151	0.41965412	2.837885536	2.757543749	1.20E-06	1.76E-06
AC068580.1	0.204722347	0.892078858	2.123502654	2.97E-13	7.71E-13
AC009065.5	0.169893115	0.610909471	1.846331221	5.94E-21	5.28E-20
NUP50-DT	1.823179193	4.173294113	1.194730233	2.48E-12	5.94E-12
Z95115.1	0.339388079	1.009343242	1.572409069	5.82E-14	1.60E-13
AC104794.3	1.431963411	3.121834393	1.124399377	4.55E-14	1.27E-13
AC008549.1	23.38094883	11.31305724	-1.047344621	3.88E-14	1.09E-13
SLC6A1-AS1	0.15158503	0.554465243	1.870969739	4.88E-06	6.81E-06
AC007298.2	1.130859474	0.532698123	-1.08602956	1.08E-12	2.68E-12
DNAJC3-DT	0.43136521	1.022507692	1.245129966	7.99E-11	1.68E-10
AC018904.1	0.950886676	2.411730255	1.342723234	4.94E-16	1.80E-15
AL049840.6	2.05730479	5.375174621	1.385556079	1.63E-19	1.02E-18

AC009237.15	0.246232376	0.665483481	1.434382388	5.71E-12	1.31E-11
AL021807.1	0.076370158	0.629068154	3.042135407	1.32E-16	5.07E-16
AC124798.1	0.181985717	0.617449882	1.762496814	0.025528244	0.028423985
HCG18	0.341766982	0.924909344	1.436298938	2.01E-18	9.85E-18
NRAV	0.820404199	2.090722421	1.349594751	3.98E-19	2.30E-18
LINC02506	0.13755911	2.400834645	4.125412452	7.54E-05	9.84E-05
AC040970.1	0.106725055	0.629852031	2.561114035	7.47E-06	1.03E-05
PITPNA-AS1	1.650809657	4.433618872	1.425310974	6.23E-16	2.24E-15
AL162582.1	0.133942952	1.827624126	3.770278813	1.23E-09	2.30E-09
