# Supplementary material for: Identification of Epithelial Mesenchymal Transition-Related lncRNAs Associated with Prognosis and Tumor Immune Microenvironment of Hepatocellular Carcinoma
Source: Dis Markers. 2022 Jan 15;2022:6335155. doi: 10.1155/2022/6335155 (PMC8802097; doi:10.1155/2022/6335155)
Supplement: Supplementary 2 — Supplementary Table 2: 492 EMT-related lncRNAs associated with OS signature screened by the univariate Cox regression analysis. [file 6335155.f2.pdf]

Id	HR	HR.95L	HR.95H	P-value
SNHG15	1.12923702	1.007351741	1.2658699	0.037009612
AL354733.3	1.101176225	0.737275989	1.644688146	0.637734684
MUC12-AS1	1.255638248	0.994295217	1.585673334	0.055887222
AC006042.1	0.98293021	0.760852547	1.269827906	0.895169596
VPS13B-DT	1.04288284	0.945220131	1.150636326	0.40260674
AP006621.2	0.98969662	0.872652971	1.122438622	0.871871436
THUMPD3-A	1.492884282	1.250498816	1.782251573	9.29E-06
AC025265.1	1.205387454	1.030712526	1.409664555	0.01935157
AC022613.1	0.973081443	0.916571357	1.033075589	0.371354033
AL451165.2	1.044013573	0.980777768	1.111326517	0.176656878
AL359513.1	1.332606109	0.962989051	1.844090584	0.083198252
LINC02428	0.958052706	0.918907946	0.998865003	0.044081047
AC005586.1	0.973649041	0.699049676	1.356116008	0.874481533
MYLK-AS1	1.357240901	1.049487149	1.75524099	0.019906296
LINC00942	1.033019339	1.001830646	1.065178988	0.037811331
LINC00886	0.864446294	0.683867802	1.092707381	0.22307318
AL355353.2	1.050609583	0.962111246	1.147248306	0.271484744
ZNF529-AS1	1.37387136	1.074661654	1.756387703	0.011259807
ZNF213-AS1	1.11269793	0.872853667	1.418447021	0.388624341
LINC00896	1.111766519	0.827140548	1.494334663	0.482562067
AC073611.1	1.92453978	1.460045474	2.536806852	3.39E-06
AL135905.1	0.967463606	0.904078569	1.035292574	0.338694938
TAT-AS1	0.846974692	0.688036571	1.042627905	0.117281459
DANCR	1.022182281	1.004663044	1.040007017	0.012867984
AL645933.3	1.009425256	0.875379887	1.163996753	0.897319172
SNHG12	1.17163711	1.066845273	1.28672222	0.000921349
LINC01139	1.004238644	0.963292061	1.04692574	0.842150039
AC093673.1	1.030074789	0.994972181	1.066415816	0.093928317
AC005586.2	1.135303134	0.912253839	1.412888771	0.255509211
LINC01942	0.858602073	0.701049953	1.051562041	0.140511892
LNCTAM34A	0.897015475	0.639737093	1.25776162	0.528572326
SNHG8	1.005553636	0.998527866	1.012628839	0.121587056
AL161669.3	0.982859143	0.865297226	1.116393381	0.7902377
TMPO-AS1	1.141564984	0.974934685	1.336674788	0.100043975
MAN1B1-DT	1.304331127	0.822408502	2.068655278	0.258861937
AL139384.1	1.222209542	1.068483502	1.398052625	0.00343563
AZIN1-AS1	1.139079188	0.819605168	1.583081036	0.438099994
AC112491.1	1.028676691	0.980256411	1.079488718	0.250417169
AC068987.3	1.15579648	1.077045048	1.240306064	5.79E-05
AC010547.2	1.002460546	0.954848078	1.052447157	0.921150302
AC016405.3	1.382242708	1.156044314	1.652700403	0.000384754
AL606489.1	1.15380122	0.97223336	1.369277491	0.10150012
ZSCAN16-AS	0.990836124	0.950585985	1.032790551	0.66349265

AC009407.1	0.999813915	0.979210894	1.020850432	0.98602367
AC090589.3	1.191495567	0.871428359	1.629120365	0.272322367
ZFPM2-AS1	1.084341408	1.036862145	1.133994808	0.000393259
AC012510.1	1.202356772	1.001296964	1.443789264	0.048399182
RAB30-DT	1.141428822	0.991897753	1.313502075	0.064832686
AC011468.1	1.079984409	0.896901656	1.30043948	0.416856583
LINC01671	1.098684121	0.90099182	1.339753337	0.352443511
AL355102.4	1.016359463	0.986912558	1.046684987	0.279362759
ZNF710-AS1	0.905470401	0.774244786	1.058937253	0.213835798
ASMTL-AS1	1.076179951	1.007268153	1.149806318	0.029671344
AC020765.2	1.215704158	0.906506167	1.630365742	0.192085095
AC015982.2	1.446721214	1.088538503	1.922763657	0.010943858
AC007099.1	1.013911571	0.989244363	1.039193866	0.271584974
AL031673.1	1.090916736	0.988860499	1.203505778	0.082487722
SNHG32	1.008479587	1.001758487	1.015245781	0.013326109
RUSC1-AS1	1.160793492	1.043242874	1.291589489	0.006198614
AC008771.1	1.118056637	1.001874014	1.247712413	0.046216697
CRIM1-DT	1.039333361	1.001212449	1.078905717	0.043019649
LINC01004	1.424151524	1.07992344	1.878103103	0.012257901
DLG5-AS1	1.079481433	0.949150344	1.227708731	0.244017867
MUC20-OT1	1.215206621	0.826570958	1.786570309	0.321546889
AP000894.4	1.131772799	0.962541246	1.330758214	0.134144249
ZEB1-AS1	1.329083311	1.019692641	1.732347942	0.035360708
ZNF503-AS2	1.401073439	1.064571847	1.84394016	0.016107492
AL445524.1	1.001909027	0.992612409	1.011292715	0.688432265
AL354836.1	1.0304711	0.910968734	1.165649983	0.633165237
AP002748.4	1.159650802	0.881749371	1.525138579	0.289304988
lnc-CCNY-1	1.655312487	1.351399952	2.027571057	1.12E-06
ZNF793-AS1	1.103499019	0.962252079	1.265479297	0.158736735
FAM99B	0.977781278	0.927051558	1.031287008	0.408460071
AC009022.1	0.904970952	0.553817818	1.478775867	0.690236224
AL162413.1	0.967300359	0.930776661	1.005257248	0.09046369
AL035071.1	1.088216425	1.005489201	1.177750081	0.036112032
AC012146.1	1.176821563	1.025533408	1.350427964	0.02039054
AC009275.1	1.166794296	1.00350723	1.356650842	0.044911707
AP001372.2	1.327786204	0.950700683	1.854438767	0.096241435
AL358472.2	1.232293485	0.856044486	1.773911587	0.261121249
AC139530.1	1.194138133	0.999210166	1.427093047	0.051024442
AC022424.1	1.049826241	1.015982813	1.084797028	0.003633023
AL354892.2	1.142111222	1.04140665	1.252553979	0.004780728
KCNMB2-AS	1.176027766	1.02636412	1.347515253	0.019561292
MID1IP1-AS	0.983182173	0.811029183	1.191877192	0.862889
NOP14-AS1	1.393445856	1.027174	1.890323696	0.032984285
C2-AS1	0.89508165	0.639177529	1.253440749	0.518830059

AC068888.1	1.215735093	1.013373283	1.458506791	0.035467839
BX322562.1	1.002306665	0.929809465	1.080456469	0.952038957
SNHG19	1.005978465	0.998809424	1.013198962	0.102364542
AL121899.1	1.014163986	0.941342043	1.092619413	0.711420451
AC007773.1	1.325745931	1.095290195	1.60469096	0.003801466
LINC02298	1.092811938	0.939157157	1.27160606	0.25096205
PARD3-AS1	1.178804311	0.892423255	1.557085829	0.246679202
AC145207.5	1.976943903	1.500703237	2.604317162	1.26E-06
AC087741.1	1.535894762	1.105172598	2.134483541	0.010603391
AC024075.1	0.937526497	0.598721037	1.46805587	0.777986094
AC022144.1	1.03602166	0.955051345	1.123856728	0.394043228
AL360181.2	1.093033138	0.805602397	1.483016243	0.567717046
AC027644.3	1.036934133	0.957105343	1.123421161	0.374897434
LINC00513	1.145630395	0.904599049	1.450884792	0.25929756
BAIAP2-DT	0.992885914	0.937501333	1.051542439	0.807390387
SNHG10	1.40913328	1.205683096	1.646914192	1.62E-05
AC004687.1	0.898144419	0.695119698	1.160466896	0.411270003
STPG3-AS1	0.773489981	0.577819062	1.035422316	0.084340676
SNHG29	1.005591762	1.000848472	1.010357532	0.020803149
AC023043.4	1.1343168	0.964111208	1.334570734	0.128673409
MAP3K2-DT	1.200197971	1.027357354	1.40211696	0.021439252
AC027307.2	1.015724372	0.972571871	1.060791528	0.4811972
KMT2E-AS1	1.020052427	0.96743094	1.075536156	0.462526861
BX537318.1	1.188811634	0.960992123	1.47063963	0.111071671
AC020915.3	1.508763763	1.180002109	1.929122054	0.001038448
AL356234.2	0.827450742	0.663496761	1.031918725	0.092744883
AL392172.1	1.031701971	0.977290687	1.089142638	0.258899131
CCDC18-AS1	1.127736189	0.89787542	1.416442508	0.301288681
AC138696.2	1.068909968	0.999431014	1.143218997	0.051971923
MINCR	1.040472983	0.964153989	1.122833115	0.307360639
RPARP-AS1	1.179408871	0.934400872	1.488660089	0.164867484
AC103691.1	1.111327132	0.891926421	1.384697173	0.346861125
LINC00685	1.14643291	0.959229445	1.37017105	0.133006608
AL139287.1	1.080158013	0.989295571	1.17936577	0.085448757
ALDH1L1-AS	0.694872003	0.471059357	1.025023902	0.066453639
AC040977.1	1.019599247	0.92675891	1.121740091	0.690286045
CYTOR	1.064436661	1.028479296	1.101651156	0.00036864
AC115618.2	1.03547667	0.957413716	1.119904506	0.383352446
MELTF-AS1	1.169853739	1.039866796	1.316089499	0.009041888
SNHG1	1.051663596	1.023521969	1.080578973	0.000272655
AC104113.1	1.45264074	1.133839894	1.861078562	0.003141121
GPRC5D-AS	1.510260104	1.095650636	2.081763572	0.01180786
LINC01857	0.892013997	0.683602063	1.163965141	0.399975768
AC010331.1	1.313163827	0.943327502	1.827996356	0.106468024

AC005332.4	1.222289163	0.942382313	1.58533408	0.130348798
AC000123.1	1.289577234	0.925744335	1.796402506	0.132648316
AL035461.3	1.099837563	1.021391926	1.184308035	0.011715347
AL022328.2	1.017636777	0.87798175	1.179505849	0.816432516
CAMTA1-DT	1.225477525	0.87795054	1.710569212	0.232094792
AL118516.1	1.085909336	1.000492445	1.17861868	0.04863904
AC132872.1	1.061881504	0.965117205	1.168347558	0.218083595
SREBF2-AS1	1.519553164	1.189027639	1.941958069	0.000827461
AC147067.1	1.061134547	0.874031683	1.288290286	0.548797441
AC091271.1	1.048122906	0.8992315	1.221667197	0.547677439
AC020978.4	0.972606355	0.912971343	1.036136708	0.389586855
AC124016.3	0.953660746	0.662084418	1.373644801	0.798846583
FAM111A-D'	1.529203101	1.029483993	2.271489541	0.035387581
AL022328.1	1.114940149	0.813683491	1.52773351	0.498404009
TMEM147-A	1.305925605	1.022840322	1.667358678	0.032264028
AC012313.1	1.237933379	0.875625847	1.750152825	0.226981156
AP000424.2	1.097870439	0.966849764	1.246646115	0.149856837
NCK1-DT	1.428112757	1.083590069	1.882174914	0.011409427
AL158071.3	1.124361736	0.827857063	1.527062303	0.45297835
AL928654.2	1.273607603	0.999442193	1.622981636	0.05052966
AC019117.2	1.022731069	0.952645279	1.097973046	0.534887424
AC068473.5	1.458537561	1.191628331	1.785230983	0.000252108
SNHG7	1.048991519	1.024162995	1.074421954	9.09E-05
CR936218.2	0.785954855	0.610551852	1.011748686	0.061579354
LINC00957	1.097108609	0.869435059	1.384401614	0.434819561
CD2BP2-DT	1.036664727	0.91767279	1.171085999	0.562688231
LINC01963	1.257479113	0.941398947	1.679685031	0.120872605
AC015813.1	1.100496062	0.930649673	1.301339933	0.262869419
AL023803.1	1.006801367	0.891984094	1.136398058	0.912632585
AC006504.7	1.616184932	1.329444369	1.964770994	1.45E-06
APTR	1.116529025	0.963735563	1.29354681	0.142102096
LINC00106	1.089469322	0.981789999	1.20895854	0.106560648
SNHG9	1.00115946	0.980112537	1.022658344	0.914871507
TAF1A-AS1	0.873701318	0.684112605	1.115830914	0.279337341
AC008267.5	1.007848997	0.957293766	1.061074079	0.765886741
AC009065.9	1.090363273	0.855038509	1.390454413	0.485535482
AC008610.1	1.166317253	1.014420807	1.340958234	0.03068915
AL161668.4	0.951347883	0.889951868	1.016979488	0.14283736
AC026462.3	1.019830383	0.990042699	1.050514297	0.19418089
EIF3J-DT	1.119118327	0.852067791	1.469866415	0.418475743
PURPL	0.941670511	0.734975446	1.206493845	0.634557444
LINC01980	1.0538563	0.991903409	1.11967868	0.089702197
MIR210HG	1.182600789	1.079430471	1.295631968	0.000316874
OTUD6B-AS	1.063746472	0.916021417	1.23529487	0.417883134

AC016394.2	1.787831153	1.382035454	2.312777305	9.72E-06
AL031058.1	1.027734115	0.999201971	1.057080992	0.056859669
AC009237.14	1.118216276	0.991813868	1.260728128	0.067901707
AC010761.1	1.659406309	1.298648567	2.120380655	5.14E-05
AP001505.1	1.017539055	0.969920344	1.06749563	0.477072899
AC132872.3	1.451287347	1.057079844	1.992503194	0.021265358
AC022150.2	1.384449621	1.055184949	1.816459527	0.018893907
AC083880.1	1.327696444	0.948673411	1.858150366	0.098385459
AC016888.1	1.01254992	0.969714799	1.057277192	0.571725365
LINC01003	1.058983995	0.905022075	1.239137843	0.474629775
AL359643.3	1.369631642	1.040920771	1.802145645	0.024679031
AC079305.2	1.030049918	1.009086195	1.051449162	0.004770402
MAPKAPK5-	1.137601716	1.055040168	1.226624071	0.000797245
AC004540.2	1.012966968	0.911506094	1.125721578	0.810905624
AP000593.3	0.990064948	0.949487601	1.032376409	0.640043985
AP000254.2	1.03134088	0.911430386	1.167027157	0.624591197
LINC02806	1.053993103	0.980741709	1.132715627	0.152475523
AC060780.1	1.000961538	0.943707739	1.061688868	0.974487212
SNHG14	1.450802779	1.218181778	1.727844515	3.00E-05
AL589765.4	1.035107076	0.912191393	1.174585364	0.592656475
AC087482.1	0.996453772	0.97669467	1.016612611	0.728107981
AC027796.4	1.157340679	1.030868751	1.299328791	0.013328414
LINC00221	1.143774482	1.050215277	1.245668477	0.002033934
AL391427.1	1.049822838	1.016310765	1.084439947	0.003309646
LINC00665	1.123461846	1.032671408	1.2222344	0.006774545
PTOV1-AS2	1.168913361	1.02870931	1.328225992	0.016658639
SNHG11	1.033147424	0.991485712	1.07655974	0.120471024
PPP1R14B-A	1.095823543	0.95260843	1.260569611	0.200356402
AL121832.2	1.055682528	0.958187571	1.163097534	0.273060708
AC005920.4	0.982624133	0.875475926	1.102886049	0.766042733
AC010542.6	1.154215124	0.874073175	1.524143047	0.311966048
HEIH	1.036042674	1.001809246	1.071445913	0.03888481
AC107375.1	1.278896271	0.991152876	1.650174974	0.05854028
AL391056.1	0.998134856	0.871795631	1.142782959	0.978430103
FOXD2-AS1	1.146201153	1.036327861	1.267723403	0.007954198
LINC01089	1.162615559	0.932204525	1.449976804	0.181219791
AL355802.3	1.62040795	1.111920465	2.361429623	0.012001375
LINC01436	1.02600633	0.979473701	1.074749622	0.27829427
MAGI2-AS3	0.866401391	0.675055773	1.111984224	0.260036891
AC092171.3	1.159670222	0.930464983	1.445336523	0.187338905
AL591895.1	1.003907796	0.986308722	1.021820897	0.665583584
AC074117.1	1.661844107	1.292561807	2.136629614	7.45E-05
LINC02163	1.104872172	0.956520333	1.276232689	0.175199526
PVT1	1.138666729	0.993486761	1.305062102	0.062032624

AC125257.1	1.091802561	0.93240314	1.278452185	0.275378676
AC008764.2	1.099380496	0.881161344	1.371641509	0.401311835
RNASEH1-A	1.160177129	1.037975766	1.296765314	0.008888311
NRSN2-AS1	1.281092958	1.046996826	1.567530223	0.016126398
AC009283.1	0.967820019	0.890967721	1.051301373	0.438433701
CHKB-DT	1.136743787	0.901385654	1.433555583	0.27888636
PANK2-AS1	1.411598181	0.977565296	2.038338955	0.065926496
LINC01484	0.998499208	0.662573696	1.504739283	0.994273114
GIHCG	1.119218642	1.001984687	1.250169174	0.046033623
SNHG17	1.058799826	1.000810257	1.120149462	0.046795449
ST8SIA6-AS	1.045058063	0.971994948	1.1236132	0.23332789
AC104825.1	1.175357068	0.946576933	1.459431546	0.143502361
LINC00294	1.187149443	0.997537117	1.41280337	0.053335961
AC006449.7	1.039909336	0.820433295	1.318097929	0.746276254
AC006213.4	1.238454763	1.033697361	1.483771032	0.020373634
AC084824.6	0.989847079	0.738275185	1.327143671	0.945619585
AC015849.3	1.04901675	0.688045144	1.599366192	0.824016131
AC111000.4	1.028595097	0.995795367	1.062475191	0.08816903
RAD51-AS1	1.159721046	0.916057999	1.468196233	0.218181992
AC024060.2	1.180038134	1.058411507	1.315641401	0.002855902
POLR2J4	1.087296164	0.784567968	1.506833056	0.615179128
MHENCR	1.07260996	0.99493274	1.15635166	0.067622448
LINC01474	1.009454591	0.999171632	1.019843378	0.071651015
AL031186.1	1.392287297	1.022916462	1.895036388	0.035377417
AC005229.4	1.184690384	0.95652435	1.46728236	0.120486239
AP000759.1	1.04515771	0.880106264	1.241162213	0.614508535
AC074212.1	0.970587798	0.738805097	1.275086863	0.83021006
BX284668.5	1.003571011	0.971991643	1.036176372	0.827026257
AL391244.2	1.258539808	0.9253073	1.711779912	0.142840218
AL355488.1	1.162759427	1.013462321	1.334050074	0.03150102
AC008915.3	1.135198146	0.971646566	1.326279407	0.11013439
AP003119.2	1.055199775	0.952071503	1.16949889	0.305855737
AL160006.1	1.170669315	0.92432002	1.482675497	0.19116335
GLIS2-AS1	0.925528719	0.815977537	1.049787979	0.228577538
AL844908.2	0.893803009	0.616232323	1.296400383	0.554025302
SBF2-AS1	1.543007713	1.157818058	2.056344505	0.003076353
MIR4458HG	1.040147115	0.899196848	1.203191518	0.596244217
AL118558.3	1.28281892	1.032217578	1.594261149	0.024710088
ASH1L-AS1	1.298486213	1.029861468	1.63717791	0.027189566
AC012645.1	0.994992058	0.665536966	1.487534497	0.980478376
AL021707.6	1.072863466	0.93319356	1.23343759	0.322989483
AC092535.5	1.020149561	0.997406555	1.043411156	0.082879249
AC009686.2	1.33864172	1.034413766	1.732345135	0.026610923
LINC01176	1.13563345	0.937105279	1.376220326	0.194505643

AP003352.1	1.123507838	0.96545959	1.307428995	0.132186965
C2CD4D-AS	1.004485395	0.943735944	1.069145364	0.888181754
AC242426.2	1.292794674	0.908984506	1.838665078	0.1530129
AC099508.2	0.724700258	0.521582448	1.006917442	0.054999182
AC026740.1	1.025890762	0.974869103	1.079582737	0.326060547
AC022784.1	0.990970053	0.923416999	1.063464987	0.801186603
LINC01006	1.060582579	0.911337731	1.234268448	0.447174963
SNHG3	1.083953878	1.046316261	1.122945378	7.79E-06
AC004918.5	1.126058268	0.874396437	1.450151407	0.357605344
AL355574.1	1.575773039	1.28509638	1.932198012	1.24E-05
SCAMP1-AS	1.138916866	0.945753419	1.371532582	0.170130628
AL512598.1	1.145056125	1.001466553	1.309233468	0.047546339
DLGAP1-AS	1.060798567	0.984104328	1.143469821	0.1231998
MIR4435-2H	1.168065716	1.059093758	1.288249985	0.001877405
AP002360.2	1.047646038	0.991149858	1.107362538	0.099832412
AC013275.1	1.006689703	0.910888511	1.112566628	0.896031392
LINC00847	1.072562486	1.001781326	1.148344711	0.044319339
VPS9D1-AS1	1.124356506	0.995686153	1.269654648	0.058725248
LINC02041	1.024908371	0.965580581	1.087881416	0.4186926
PRRT3-AS1	1.207554936	1.107022142	1.317217486	2.11E-05
AC010883.1	1.022594036	0.863694861	1.210726855	0.795401548
PSORS1C3	1.034743165	0.85498963	1.252288192	0.725739329
AC106820.3	1.145127741	0.98197254	1.335391256	0.083989142
AC136475.2	1.007044509	0.839129287	1.208560658	0.939875338
AC026471.4	0.930176819	0.755722324	1.144903209	0.494595375
AC016773.2	1.599621202	1.156517464	2.212494035	0.004530486
LINC02604	1.080973207	0.904059503	1.292506822	0.393173911
AC007038.1	1.564542648	1.163181323	2.104395635	0.003082449
AC010326.3	1.102417891	1.014636465	1.197793741	0.021268363
AC243964.3	0.973607631	0.888558285	1.066797569	0.566303274
ARRDC1-AS	1.162787058	1.048456348	1.289585155	0.004289766
MMP25-AS1	0.920576738	0.63055494	1.343993167	0.668186679
GAS5	1.008344628	1.003013168	1.013704427	0.002124269
LINC01186	1.087451574	0.853328575	1.385809594	0.497933942
AC132192.2	1.255802008	1.053094566	1.497528081	0.011214727
AC007405.4	1.134509474	1.011257832	1.272782969	0.031495774
HCP5	0.981791358	0.952589748	1.011888142	0.232932041
AP002761.4	1.135140967	0.909946517	1.416066759	0.261218573
AC009779.3	1.149872136	1.040853729	1.270309067	0.005999015
IDH1-AS1	1.152402352	0.958462974	1.385584229	0.131367657
AL162595.1	0.887500062	0.578776751	1.36089841	0.584254647
LINC00853	1.026465379	0.932766601	1.129576438	0.5927534
LENG8-AS1	1.291218109	1.032783343	1.614321353	0.024892955
AC008443.5	1.094663343	0.982778076	1.219286291	0.100140817

AC005261.3	1.104874276	0.966562885	1.262977489	0.143860738
AC022306.2	0.990274479	0.755549833	1.297920403	0.94355402
AC142472.1	1.192702271	0.856365278	1.661135434	0.29713981
SNHG21	1.788248149	1.251942902	2.554294958	0.001397522
AC011445.2	1.012703939	0.986535464	1.03956655	0.344612056
AC005840.4	0.987298465	0.660072636	1.476743931	0.950382039
NEAT1	1.00094027	0.981000932	1.021284886	0.927059969
AC016394.3	1.389967687	1.177461593	1.640826488	0.000100367
AC012313.9	0.943387547	0.804154837	1.106727241	0.474423408
AC103706.1	1.003020125	0.869308629	1.157298269	0.967048285
ARHGAP27P	1.221315203	0.892369776	1.671516524	0.211767255
AC068580.3	1.256025443	1.062937272	1.484189101	0.007435961
AL590666.2	1.002093659	0.917562432	1.094412399	0.962899594
PRR34-AS1	1.031055461	0.983021199	1.081436865	0.208957644
ST7-AS1	1.110144521	0.667834376	1.845398951	0.686962248
TBX2-AS1	1.071259893	0.88695829	1.293857639	0.474844268
AC005332.5	1.26550482	1.103070225	1.451859014	0.000780673
AL359504.1	1.428505545	1.050851628	1.941880315	0.022810142
LINC02035	1.205350358	0.898765115	1.616517443	0.212317877
LINC02027	1.092280358	1.014032693	1.176565991	0.019944006
AP003486.1	0.845463704	0.55953265	1.27751057	0.425407561
AC012467.2	1.904504388	1.384496392	2.619824062	7.51E-05
AC006026.3	1.245057405	0.918639524	1.687460535	0.15767972
AC010973.2	1.760627647	1.259557559	2.461030615	0.000931548
ITGB1-DT	1.00904503	0.98522478	1.033441193	0.460068319
AL365181.3	0.998676403	0.954566965	1.044824087	0.954173825
AC011700.1	1.308912935	0.965467692	1.774531748	0.082981369
AC008735.2	1.177152203	1.067375098	1.298219634	0.001093178
AC068506.1	1.247855856	1.11435959	1.397344493	0.000125248
AC011477.2	0.986721347	0.902441919	1.078871667	0.769179739
AL121845.4	0.987615763	0.912677563	1.068706994	0.75692929
U91328.1	0.995444017	0.750655275	1.320058384	0.974703146
AC005332.3	1.059492072	0.998674886	1.124012896	0.055364965
AL050341.2	1.115094696	1.004772674	1.237529855	0.040409184
AC005261.1	1.062599382	0.975721556	1.157212771	0.162954947
AC139100.2	1.285177206	1.037266027	1.592340255	0.021756922
AC012615.1	1.229250379	1.005169479	1.503285293	0.04441214
ASB16-AS1	1.083369543	0.944381453	1.24281302	0.253005295
AL109615.3	0.947435739	0.811469278	1.106184181	0.49450387
LINC01549	0.980164401	0.930967662	1.031960929	0.445735177
AC087741.2	1.212177124	1.064550894	1.38027537	0.003683882
ZNF687-AS1	1.045416902	0.967832648	1.129220534	0.258930625
AC007566.1	1.071160931	0.76291293	1.503953721	0.691345327
Z98257.1	1.03260548	0.952672173	1.119245535	0.435087566

AJ009632.2	0.992085069	0.931553504	1.056549924	0.804603915
CEBPA-DT	1.068916146	1.02385867	1.115956489	0.002421188
AC026369.2	1.374040827	1.120874037	1.684389263	0.002226838
AL162411.1	1.099497743	1.034473577	1.16860915	0.002291046
AC008608.2	1.1117	0.978208295	1.263408721	0.104719394
ARHGAP5-A	1.429376381	1.042976541	1.958928853	0.026307
AC010719.1	1.109090179	1.002292457	1.227267567	0.045039033
AL441992.2	1.067218196	0.993050725	1.146924976	0.07669237
LINC00205	1.281504141	1.130896911	1.452168494	0.000100916
TRIM52-AS1	1.089520529	1.01870157	1.16526274	0.012408559
AC064807.4	0.790992913	0.52638508	1.188616114	0.2591533
AC078993.1	0.95467853	0.857089644	1.063378962	0.399218964
AP000240.1	1.309096318	1.069030201	1.603072737	0.009166726
SH3BP5-AS1	1.124449268	0.906392192	1.394965852	0.286242966
AC023509.3	0.997429457	0.880465028	1.129931899	0.967738963
AC002456.1	1.049178239	0.919224785	1.197503587	0.476729944
AC127024.5	1.238913439	1.01216736	1.516455252	0.037780598
AC015912.3	1.128322653	1.036197885	1.228637915	0.005466054
AC099850.4	1.131391178	1.068691185	1.197769772	2.20E-05
AC244090.1	1.009562116	0.983255679	1.036572366	0.479905463
AC084018.1	1.240854143	1.017363737	1.51344003	0.033182605
LINC01093	0.994212788	0.976609026	1.012133866	0.524279545
AC010531.6	1.222690564	1.018500622	1.467816693	0.031037084
ZNNT1	1.106864059	0.96745343	1.266363844	0.139347817
AC110285.2	1.00994267	0.96470917	1.057297088	0.672163786
AL365330.1	1.410689948	1.066779748	1.865470481	0.015805188
ELFN1-AS1	1.056397587	1.019579236	1.094545498	0.002435361
AC008736.1	0.978719154	0.891934335	1.073948098	0.649790132
AL031985.3	2.316110412	1.740326504	3.082391396	8.44E-09
AC109322.1	1.214451647	1.015542673	1.452319871	0.033257327
AL135999.1	1.125935988	0.807990687	1.568993145	0.483537987
AL365203.2	1.1359493	1.063183696	1.213695072	0.000160726
LINC00997	1.224510503	0.888611631	1.687380538	0.215686198
AC010280.2	0.837355864	0.696620241	1.006523787	0.058664171
AC084125.4	0.885271995	0.566928117	1.382373676	0.592009998
AC253536.6	0.951939981	0.854396916	1.060619146	0.37187746
AC007996.1	1.184889395	0.917978899	1.529406482	0.192652773
LINC02241	0.922921298	0.787225659	1.082007062	0.322873667
U62317.2	1.031182455	0.984326567	1.080268776	0.195612291
HAGLR	1.021865457	0.96441753	1.082735414	0.463750932
CTBP1-DT	1.12537997	0.894740308	1.415472249	0.312753438
AC005696.1	1.109337852	0.927886968	1.326271963	0.254850117
MAFG-DT	1.173750868	1.079572569	1.276144965	0.000173926
AC015871.3	0.933584835	0.62459277	1.395438252	0.737534509

AC023090.1	1.092007512	0.991416165	1.202805085	0.074241761
AC007541.1	1.189621926	0.932726763	1.517272136	0.161846563
AC064836.2	1.311341818	1.068432094	1.609477452	0.009507325
AC007406.2	1.03122932	0.954386301	1.114259404	0.4363788
AC095057.3	1.530872799	1.172529342	1.998731667	0.001749511
LUCAT1	1.281756393	1.113603074	1.475300752	0.000540993
ZFAS1	1.009737212	1.001908075	1.017627528	0.014689017
SNHG30	1.129756162	1.050784015	1.214663498	0.000967617
LINC01703	1.032543842	0.86258917	1.235984432	0.727073061
AC004816.1	1.215312057	1.067590531	1.383473675	0.003186902
AC004148.1	1.213461421	0.90820364	1.621319885	0.190642739
WNT5A-AS1	1.159057805	0.98393766	1.365345642	0.077360875
AC024075.2	1.131672564	0.979102838	1.308016628	0.094103102
AL117379.1	1.280030237	0.933484524	1.755227179	0.125359626
AC084824.5	1.425573275	1.091697194	1.86155939	0.009204235
AC002398.1	1.152870441	0.972734222	1.366365265	0.100785516
AC048341.2	1.159327023	1.008252308	1.333038503	0.037955757
AL133353.1	1.023794046	0.804042835	1.303604986	0.848719385
AC016747.1	1.159480133	1.017405478	1.321394672	0.026507304
AC104958.2	1.016281664	1.007152879	1.025493192	0.000451232
YTHDF3-AS	0.982248515	0.86066456	1.121008335	0.790497579
AL390728.6	1.038190708	1.003502358	1.07407814	0.030648442
DSCR8	1.049702626	1.001975893	1.099702708	0.041041917
LINC00623	1.115191031	0.912330571	1.363158351	0.287197753
LINC01770	0.968007602	0.883201579	1.060956795	0.48700888
AC090587.1	1.086844533	0.866851971	1.362667536	0.470480922
BACE1-AS	1.254124339	1.131598085	1.389917391	1.58E-05
AP003119.1	1.244140419	1.076303336	1.438149758	0.003131762
SNHG25	1.014902861	0.995231899	1.034962622	0.138513712
AC120053.1	1.142063646	0.977807678	1.333911976	0.093602512
LINC00342	1.125287129	0.967035937	1.309435435	0.126889299
AL603839.3	1.316163105	1.036899123	1.670640162	0.023961048
AL359921.2	1.210090503	1.045496924	1.400596207	0.010575595
PRANCR	1.479212962	1.103212272	1.983363529	0.008886453
LINC02062	1.445738558	0.82760417	2.525555156	0.195271415
ANKRD10-IT	1.074638298	1.006036979	1.147917518	0.032452218
AC011477.1	1.008459905	0.906634335	1.121721671	0.876724252
AC084036.1	1.06199911	0.994144154	1.134485481	0.074160124
AC083843.3	1.05975062	0.790797034	1.420176516	0.697617311
AFDN-DT	1.375895105	1.005489118	1.882752687	0.046133128
AC131009.3	1.13952284	0.996161847	1.303515394	0.056922263
LMNTD2-AS	1.072328406	0.913652911	1.258561317	0.392717532
AC090015.1	1.001549333	0.876978353	1.143815082	0.981773979
AL360181.1	0.978162792	0.924124753	1.035360696	0.446368286

SNHG20	1.349153861	1.09520734	1.661983145	0.004881921
AC092171.5	1.055948103	0.920670157	1.211103008	0.436394422
LINC01270	1.085358102	0.883402828	1.333482498	0.435530622
AC112206.2	0.990121667	0.906545502	1.081402879	0.825373224
AL121944.2	1.056741971	0.927303188	1.20424863	0.407754261
LINC01836	1.053141796	0.951746104	1.165339829	0.316126778
AC055822.1	1.300123339	0.912865322	1.851664921	0.145759672
AC009005.1	1.188666288	1.076401876	1.312639429	0.000639027
TMEM161B-.	1.225134748	0.967188019	1.551875252	0.092302315
AC010969.2	1.518666777	1.182268247	1.950782984	0.001073414
LINC01503	1.011171482	0.962623442	1.062167947	0.658095233
AL132989.1	1.064012947	0.769657531	1.47094455	0.707282661
LINC01124	1.004587297	0.955502519	1.056193592	0.85788395
AC092171.2	1.064162647	0.984600019	1.150154496	0.116759497
PXN-AS1	1.332207535	1.103866995	1.607781485	0.00278893
SNHG6	1.005056627	1.00002667	1.010111884	0.048794552
AC007448.4	1.121357511	0.881729265	1.426109712	0.350408742
ELF3-AS1	1.04977083	0.96751314	1.139022046	0.243338609
LINC01138	1.414280754	1.19593717	1.67248757	5.09E-05
AC010735.2	1.007326979	0.789813264	1.284743734	0.953096246
COA6-AS1	1.065361002	0.976122646	1.16275764	0.156041006
AC021078.1	1.378170568	0.977877497	1.942323165	0.066924551
NIFK-AS1	1.358905063	1.119206952	1.649938794	0.001952187
LINC01287	1.013121697	0.992166942	1.03451902	0.221514517
AC026401.3	1.082952602	1.026868899	1.142099386	0.003311785
TYMSOS	1.040310803	0.965009649	1.12148782	0.302597638
PTOV1-AS1	1.260467327	1.044249926	1.521453669	0.015911961
TMEM44-AS	1.027793172	0.986942804	1.07033437	0.185235399
UBR5-AS1	1.232236583	1.00282435	1.514130562	0.04694493
PRKAR1B-A	1.13964496	1.003018675	1.294881808	0.044832308
MALAT1	1.02808187	0.980570186	1.077895644	0.251298401
SLC25A25-A	1.071875866	0.962782964	1.193330081	0.205005551
LINC01151	0.976899458	0.91827141	1.039270678	0.459219273
AC068580.1	1.355687306	1.148834532	1.599784843	0.000315158
AC009065.5	1.132697981	0.741683748	1.72985416	0.564108029
NUP50-DT	1.020122142	0.946187468	1.09983404	0.603768556
Z95115.1	1.234150453	1.060586109	1.436118508	0.006515638
AC104794.3	1.064223077	0.986913811	1.147588314	0.105741652
AC008549.1	0.977513973	0.958725209	0.996670953	0.021635027
SLC6A1-AS1	0.949782694	0.683882448	1.319067581	0.758500167
AC007298.2	0.962870927	0.73424717	1.262681643	0.784416801
DNAJC3-DT	1.234812317	0.974928391	1.563972772	0.080227399
AC018904.1	1.071307412	0.972244219	1.18046428	0.164113267
AL049840.6	1.132539979	1.065994158	1.203239994	5.61E-05

AC009237.15	1.251132728	0.94995255	1.647801359	0.110811774
AL021807.1	1.054808463	0.792415481	1.404087781	0.714637371
AC124798.1	1.270448485	1.127400055	1.431647395	8.59E-05
HCG18	1.355818916	1.005307208	1.828540489	0.046081281
NRAV	1.261006079	1.122923168	1.416068683	8.88E-05
LINC02506	1.017785003	0.974447745	1.063049629	0.427165608
AC040970.1	1.267648874	1.053626923	1.525144843	0.011949852
PITPNA-AS1	1.100883236	1.026204587	1.180996378	0.00732497
AL162582.1	1.014892485	0.977076587	1.054171975	0.445460524
