# Supplementary material for: Identification of Epithelial Mesenchymal Transition-Related lncRNAs Associated with Prognosis and Tumor Immune Microenvironment of Hepatocellular Carcinoma
Source: Dis Markers. 2022 Jan 15;2022:6335155. doi: 10.1155/2022/6335155 (PMC8802097; doi:10.1155/2022/6335155)
Supplement: Supplementary 3 — Supplementary Table 3: 480 EMT-related lncRNAs associated with RFS signature screened by the univariate Cox regression analysis. [file 6335155.f3.pdf]

Id	HR	HR.95L	HR.95H	P-value
SNHG15	1.074725699	0.975636892	1.183878282	0.144234589
AL354733.3	1.046930907	0.757986802	1.446020327	0.780753323
MUC12-AS1	1.184441727	0.966961385	1.450835811	0.101969997
AC006042.1	1.0999907	0.928476411	1.303188241	0.270498368
VPS13B-DT	1.067378406	0.982212007	1.159929478	0.124312835
AP006621.2	1.0546918	0.967343055	1.149927926	0.227345928
THUMPD3-AS	1.349839382	1.160812116	1.569647949	9.73E-05
AC025265.1	1.287555127	1.115988131	1.48549806	0.000532174
AC022613.1	0.986346139	0.939634862	1.035379535	0.578625746
AL451165.2	1.04777721	0.987761267	1.111439695	0.120953053
AL359513.1	1.463920306	1.140860726	1.878461248	0.002736547
LINC02428	0.994955868	0.966913706	1.0238113	0.728830256
AC005586.1	1.048879473	0.813270667	1.352745396	0.713137072
MYLK-AS1	1.333356346	1.081456693	1.64393004	0.007082043
LINC00942	1.02329824	0.994897901	1.052509295	0.108765091
LINC00886	1.001555432	0.876441888	1.144529143	0.98178703
AL355353.2	1.110140363	1.031771431	1.194461862	0.005152877
ZNF529-AS1	1.401313853	1.153539645	1.702308649	0.000676947
ZNF213-AS1	1.196990034	0.981940761	1.459136027	0.07514202
LINC00896	1.301887673	1.021703442	1.658907511	0.032874429
AC073611.1	1.630015743	1.27619814	2.081926967	9.10E-05
AL135905.1	1.02753515	0.997240094	1.058750537	0.075246105
TAT-AS1	0.984305407	0.881365044	1.099268846	0.778958256
DANCR	1.016095029	1.001676306	1.030721304	0.028549213
AL645933.3	1.00918613	0.903400548	1.127358897	0.871423385
SNHG12	1.100829249	1.016704582	1.191914599	0.01786495
LINC01139	1.021137589	0.994511052	1.048477013	0.120742055
AC093673.1	1.022096935	0.993830922	1.051166875	0.126640049
AC005586.2	1.049573129	0.852245022	1.292590423	0.648869068
LINC01942	0.948864107	0.8232417	1.09365584	0.468812919
LNCTAM34A	0.882877418	0.681837393	1.143194173	0.344723778
SNHG8	1.005639221	1.000019245	1.011290781	0.04921844
AL161669.3	1.058465128	0.98304244	1.139674527	0.13193847
TMPO-AS1	1.153000696	1.019577553	1.303883751	0.023270582
MAN1B1-DT	1.113381995	0.74987224	1.653107557	0.594324756
AL139384.1	1.189134396	1.070639807	1.320743543	0.001218841
AZIN1-AS1	1.217599845	0.939203097	1.578518414	0.137169514
AC112491.1	1.049779324	1.018975273	1.081514595	0.001388586
AC068987.3	1.106652672	1.035236792	1.182995182	0.002906888
AC010547.2	0.998694542	0.963128609	1.035573835	0.943711187
AC016405.3	1.483571512	1.217636949	1.807586764	9.09E-05
AL606489.1	1.128339905	0.983206504	1.294896785	0.085637361
ZSCAN16-AS1	1.018119604	0.987397717	1.049797371	0.25068025

AC009407.1	1.006191006	0.991198104	1.021410691	0.420379593
AC090589.3	1.039672393	0.796892705	1.356416841	0.774317754
ZFPM2-AS1	1.124656613	1.07516917	1.176421844	3.11E-07
AC012510.1	1.241528123	1.064402397	1.448129095	0.005875187
RAB30-DT	1.07840053	0.959321925	1.212260109	0.206111896
AC011468.1	1.201078763	1.039278243	1.388069272	0.013071011
LINC01671	1.150339935	1.005508231	1.316032952	0.041353055
AL355102.4	1.008810483	0.984696078	1.03351543	0.477325706
ZNF710-AS1	0.960829178	0.877984545	1.051490843	0.38507884
ASMTL-AS1	1.034857077	0.977523002	1.095553934	0.23870795
AC020765.2	1.696661349	1.274740579	2.258231815	0.000290136
AC015982.2	1.183569435	0.900526651	1.555574848	0.226817541
AC007099.1	1.020181965	0.994803411	1.046207954	0.120042302
AL031673.1	1.113559531	1.022100616	1.213202311	0.013897887
SNHG32	1.007395572	1.00172245	1.013100822	0.01055072
RUSC1-AS1	1.124370312	1.024305482	1.234210516	0.01370355
AC008771.1	1.072146358	0.981161684	1.171568185	0.123648259
CRIM1-DT	0.990593205	0.949172197	1.033821788	0.664518224
LINC01004	1.168002748	0.915718932	1.489791651	0.21100431
DLG5-AS1	1.04729626	0.94757105	1.15751685	0.365388352
MUC20-OT1	1.253637925	0.912904448	1.721547144	0.162453863
AP000894.4	1.142160306	1.010068759	1.2915261	0.034028834
ZEB1-AS1	1.206326234	0.962464094	1.511976386	0.103539163
ZNF503-AS2	1.321310241	1.031975986	1.691764902	0.027134765
AL445524.1	1.002049825	0.995063714	1.009084984	0.566196916
AL354836.1	0.993024681	0.897824276	1.09831962	0.891718989
AP002748.4	1.275830276	1.01383064	1.605537287	0.037793626
lnc-CCNY-1	1.664242177	1.348328568	2.054174397	2.11E-06
ZNF793-AS1	1.119976507	0.996757154	1.258428265	0.05673527
FAM99B	0.979677551	0.939864712	1.021176869	0.332062701
AC009022.1	0.966547712	0.669453016	1.395489238	0.855916204
AL162413.1	1.007254299	0.990715689	1.024068997	0.39216207
AL035071.1	1.073201239	1.00910374	1.141370161	0.024551325
AC012146.1	1.184235891	1.049876383	1.335790259	0.005920998
AC009275.1	1.173557405	1.025371232	1.343159375	0.020138607
AP001372.2	1.153972111	0.886898384	1.501470358	0.286289584
AL358472.2	1.386160443	1.058109755	1.815918211	0.017792533
AC139530.1	1.245641733	1.074417979	1.444152421	0.003598602
AC022424.1	1.031963479	0.997810969	1.067284941	0.066900678
AL354892.2	1.105128154	1.017736893	1.200023547	0.017394672
KCNMB2-AS1	1.21018977	1.074196844	1.363399351	0.001708146
MID1IP1-AS1	0.917377387	0.781377375	1.077048423	0.292183897
NOP14-AS1	1.054780821	0.798841596	1.392719891	0.706834725
C2-AS1	0.868863107	0.659488252	1.144710459	0.317679036

AC068888.1	1.018514712	0.85500945	1.213287429	0.837198199
BX322562.1	1.023027034	0.968803671	1.080285246	0.412594969
SNHG19	1.004352838	0.998516774	1.010223013	0.144081667
AL121899.1	1.036313585	0.976634546	1.099639421	0.238518963
AC007773.1	1.371982741	1.14944273	1.637608026	0.000461101
LINC02298	1.013659526	0.869954128	1.181103234	0.861919267
PARD3-AS1	1.376293173	1.07742671	1.758061946	0.010557919
AC145207.5	1.607560303	1.271640562	2.032217439	7.21E-05
AC087741.1	1.409133952	1.074931217	1.847242375	0.013024746
AC024075.1	0.891836054	0.634309086	1.253917946	0.510251472
AC022144.1	1.074382649	1.005787072	1.147656505	0.033057292
AL360181.2	1.023648034	0.80208757	1.306410093	0.851022486
AC027644.3	1.076994575	1.011653154	1.146556317	0.020191344
LINC00513	1.186468862	1.028805928	1.368293399	0.018756332
BAIAP2-DT	1.019635872	0.982093116	1.058613786	0.309659031
SNHG10	1.256979927	1.085253837	1.455879244	0.002276607
AC004687.1	1.040425795	0.869988873	1.244252506	0.664171801
STPG3-AS1	0.986030636	0.815671447	1.191970638	0.884421591
SNHG29	1.004020853	0.999862565	1.008196435	0.058085085
AC023043.4	1.010809596	0.866660449	1.178934656	0.891061813
MAP3K2-DT	1.162728376	1.028204329	1.314852737	0.016246509
AC027307.2	1.016841972	0.980261738	1.054787264	0.371600185
KMT2E-AS1	1.039611496	0.997038459	1.08400238	0.06861639
BX537318.1	1.167746397	0.979022996	1.392849456	0.084663575
AC020915.3	1.504521054	1.2282934	1.842868814	7.92E-05
AL356234.2	0.920044424	0.803392506	1.053634104	0.228322505
AL392172.1	1.071106609	1.029000315	1.114935876	0.000787685
CCDC18-AS1	1.189324991	0.99715399	1.418531086	0.05382184
AC138696.2	1.082432574	1.017383687	1.15164052	0.012245598
MINCR	1.087581945	1.026674335	1.152102909	0.004300663
RPARP-AS1	1.330041212	1.099528682	1.608879927	0.003313651
AC103691.1	1.281221314	1.072621977	1.530388235	0.006272711
LINC00685	1.17730767	1.015162854	1.365350736	0.030849013
AL139287.1	1.086849079	1.012269839	1.166922963	0.021664531
ALDH1L1-AS2	1.062987486	0.828992283	1.363031258	0.630141592
AC040977.1	1.017867628	0.944294577	1.097172993	0.643620165
CYTOR	1.025168081	0.991808032	1.059650214	0.140850547
AC115618.2	1.002228711	0.941309159	1.067090849	0.944528219
MELTF-AS1	1.10965908	1.004030628	1.226400111	0.041471971
SNHG1	1.02923963	1.005320127	1.053728249	0.016295365
AC104113.1	1.586190597	1.310568441	1.919778114	2.17E-06
GPRC5D-AS1	0.920344225	0.661930307	1.279641504	0.621572908
LINC01857	1.049504445	0.941589859	1.169787004	0.382773126
AC010331.1	1.406534205	1.050436768	1.88334846	0.022002042

AC005332.4	1.437382925	1.134365724	1.82134353	0.002667369
AC000123.1	1.207009566	0.928563376	1.568952782	0.159703709
AL035461.3	1.085585766	1.025442647	1.149256332	0.004743662
AL022328.2	1.090127136	0.972523844	1.221951709	0.138442777
CAMTA1-DT	1.284079833	0.959051213	1.719262742	0.093116948
AL118516.1	1.077742247	1.009538204	1.150554131	0.024796017
AC132872.1	1.133510403	1.048298986	1.225648265	0.001672673
SREBF2-AS1	1.331887398	1.093063743	1.622891668	0.004475674
AC147067.1	1.036296054	0.875582789	1.226508247	0.678388714
AC091271.1	1.100618961	0.975647118	1.241598601	0.11898573
AC020978.4	1.000606506	0.960120072	1.042800176	0.977046581
AC124016.3	1.326005306	1.043436427	1.685095541	0.021015521
FAM111A-DT	1.186802209	0.849339083	1.658347662	0.31570896
AL022328.1	1.256113613	0.953043515	1.655560722	0.105539149
TMEM147-AS	1.290308125	1.036247862	1.606657169	0.02271301
AC012313.1	1.098554525	0.822314301	1.467592188	0.524722431
AP000424.2	0.938177413	0.813856335	1.081489227	0.37893217
NCK1-DT	1.191107955	0.937677903	1.513033587	0.15192267
AL158071.3	1.199463959	0.931780718	1.544047608	0.158075876
AL928654.2	1.087565953	0.886345513	1.334467975	0.421304573
AC019117.2	1.01923753	0.963601517	1.078085831	0.505836047
AC068473.5	1.240211774	1.037604367	1.482381236	0.018001182
SNHG7	1.030097024	1.00829984	1.052365415	0.006579095
CR936218.2	0.88377659	0.737720681	1.058749038	0.180066953
LINC00957	1.02297161	0.809742706	1.292349912	0.848968567
CD2BP2-DT	1.09859138	1.010602521	1.194241055	0.027273843
LINC01963	1.015279406	0.78446758	1.314002386	0.908259178
AC015813.1	1.021051338	0.879287415	1.18567128	0.784726947
AL023803.1	1.079967456	0.988046535	1.180440055	0.090073871
AC006504.7	1.445341015	1.211911759	1.723731645	4.16E-05
APTR	1.083748818	0.961456132	1.221596556	0.187993559
LINC00106	1.028269031	0.940622409	1.124082512	0.539689118
SNHG9	1.012768413	0.997166422	1.028614517	0.109214557
TAF1A-AS1	0.983680273	0.832780533	1.161923029	0.846443973
AC008267.5	1.010176085	0.971419686	1.050478734	0.611985454
AC009065.9	1.119581501	0.922469737	1.358811772	0.252954514
AC008610.1	1.130695311	1.000870864	1.277359479	0.048387099
AL161668.4	0.995523826	0.956488936	1.036151753	0.826009985
AC026462.3	0.999176767	0.969714838	1.02953381	0.956989194
EIF3J-DT	1.03202119	0.823744262	1.292959216	0.784040433
PURPL	1.044217138	0.872122723	1.250270636	0.637723822
LINC01980	1.058634669	1.005629125	1.114434074	0.029693989
MIR210HG	1.117498576	1.027999657	1.214789382	0.009098577
OTUD6B-AS1	1.074757995	0.955508548	1.20889002	0.229558293

AC016394.2	1.703897466	1.340242822	2.166224304	1.36E-05
AL031058.1	1.023865259	0.996999672	1.051454777	0.08212737
AC009237.14	1.042938292	0.942204993	1.154441219	0.417230173
AC010761.1	1.540094729	1.257929097	1.885552835	2.89E-05
AP001505.1	1.068203764	1.03390591	1.103639385	7.42E-05
AC132872.3	1.232151877	0.922211783	1.646257699	0.157899327
AC022150.2	1.110806698	0.866975538	1.423213766	0.405932175
AC083880.1	1.169817256	0.893525862	1.53154203	0.253871914
AC016888.1	1.00285273	0.969007668	1.037879917	0.870810477
LINC01003	1.045982954	0.922768337	1.185650066	0.482034173
AL359643.3	1.238560445	0.982548492	1.561278644	0.070149473
AC079305.2	0.98811974	0.900227149	1.084593617	0.801465929
MAPKAPK5-A	1.119423187	1.045588663	1.198471555	0.001193214
AC004540.2	1.003355922	0.903028755	1.114829512	0.950300526
AP000593.3	0.966113082	0.919940301	1.014603323	0.167668085
AP000254.2	1.103605804	1.004505077	1.21248344	0.040014479
LINC02806	0.952260799	0.868604412	1.043974238	0.297103426
AC060780.1	0.955773875	0.826570407	1.105173488	0.541575628
SNHG14	1.108795347	0.928477052	1.324133018	0.254093575
AL589765.4	1.047908116	0.92802107	1.183282854	0.45030703
AC087482.1	1.002893662	0.988417012	1.017582341	0.696910213
AC027796.4	1.013152653	0.865260529	1.186322807	0.87106411
LINC00221	1.057955952	0.975266503	1.147656352	0.17484053
AL391427.1	1.060484103	1.026648046	1.095435321	0.000385846
LINC00665	1.088032401	1.00550638	1.17733167	0.036045962
PTOV1-AS2	1.126238299	1.007898622	1.258472507	0.035828785
SNHG11	1.045861466	1.020299877	1.07206345	0.000382626
PPP1R14B-AS	1.036507805	0.911455867	1.178716897	0.584638522
AL121832.2	1.066993369	0.989769608	1.150242279	0.090704144
AC005920.4	0.983670365	0.900380297	1.074665216	0.715306944
AC010542.6	1.191006109	0.955280528	1.484899473	0.120329415
HEIH	1.026465379	0.998355675	1.055366539	0.065212056
AC107375.1	1.373289045	1.136829261	1.658932317	0.001001484
AL391056.1	1.1333865	1.042557194	1.232129005	0.003305192
FOXD2-AS1	1.116773305	1.026110012	1.215447271	0.010569467
LINC01089	1.275907173	1.071600886	1.519165518	0.006206385
AL355802.3	1.577144137	1.127283078	2.206529733	0.007831478
LINC01436	1.054025331	1.012427631	1.097332159	0.010432199
MAGI2-AS3	0.85686371	0.707724044	1.037431784	0.113350434
AC092171.3	1.092535855	0.906066413	1.317380909	0.353990154
AL591895.1	1.009203948	0.996963879	1.021594294	0.141138556
AC074117.1	1.279242032	1.031940339	1.585808903	0.024651962
LINC02163	1.04952296	0.928349862	1.186512207	0.439991178
PVT1	1.254859816	1.106642136	1.422928972	0.000400085

AC125257.1	1.14736112	1.011999315	1.300828488	0.031857799
AC008764.2	0.973399284	0.812760508	1.165787653	0.769532448
RNASEH1-AS	1.034287851	0.930825727	1.149249885	0.530702723
NRSN2-AS1	1.019195589	0.832248039	1.248137094	0.854088361
AC009283.1	1.086629227	1.025947586	1.150900001	0.004601365
CHKB-DT	1.29413229	1.100413473	1.521953726	0.001829977
PANK2-AS1	1.28169379	0.938918004	1.749608553	0.118046468
LINC01484	1.32248512	0.968901703	1.805102506	0.078249701
GIHCG	1.128602996	1.031596057	1.234732058	0.008331034
SNHG17	1.052435302	1.003723689	1.103510934	0.034542368
ST8SIA6-AS1	1.059780337	0.991279852	1.133014417	0.088556266
AC104825.1	1.069778959	0.88456546	1.293773127	0.486802925
LINC00294	1.168918652	1.02376448	1.334653469	0.021047131
AC006449.7	1.167081178	0.963591149	1.413543989	0.113978626
AC006213.4	1.295084338	1.131794377	1.481933004	0.000169619
AC084824.6	1.218590452	0.99015153	1.499732762	0.06196871
AC015849.3	1.153002606	0.846432685	1.570609256	0.366651047
AC111000.4	0.992290791	0.940575384	1.046849652	0.776877392
RAD51-AS1	1.148899787	0.948216564	1.392056173	0.156451313
AC024060.2	1.160732721	1.057019863	1.274621695	0.001801318
POLR2J4	1.158924744	0.905172498	1.483812827	0.242087188
MHENCR	1.091950923	1.030794861	1.156735315	0.00277724
LINC01474	1.00408561	0.992861985	1.01543611	0.477136319
AL031186.1	1.131300851	0.863638767	1.481917746	0.370440602
AC005229.4	1.167386833	0.991521982	1.374444583	0.063202568
AP000759.1	0.90870651	0.773715907	1.067248991	0.24331202
AC074212.1	1.04318128	0.866961053	1.255220381	0.654304536
BX284668.5	1.020175856	1.000973285	1.039746806	0.039369514
AL391244.2	1.25896051	0.983882293	1.610946326	0.067132811
AL355488.1	1.072989299	0.953306822	1.20769726	0.243009109
AC008915.3	1.128610315	0.988298846	1.288842185	0.074067674
AP003119.2	1.07802749	0.988072894	1.176171592	0.091016502
AL160006.1	0.991501851	0.798304488	1.231454834	0.938480481
GLIS2-AS1	1.017471634	0.932818073	1.109807536	0.695937482
AL844908.2	1.102059761	0.843961937	1.439088262	0.475330633
SBF2-AS1	1.301823631	1.018616243	1.66377159	0.035089084
MIR4458HG	1.156908783	1.036010189	1.291915801	0.009648837
AL118558.3	1.122618706	0.930101661	1.354983882	0.228190477
ASH1L-AS1	1.05630276	0.843027516	1.323533928	0.634061114
AC012645.1	1.251717702	0.919784152	1.703440097	0.153263066
AL021707.6	1.070725203	0.956728427	1.198305003	0.234130512
AC092535.5	1.0143297	0.993650795	1.035438954	0.175776269
AC009686.2	1.221594277	0.984215868	1.516224871	0.069425228
LINC01176	1.081106755	0.932554233	1.253323156	0.301106283

AP003352.1	1.297936905	1.155072095	1.458471913	1.17E-05
C2CD4D-AS1	1.028179862	0.981163484	1.077449217	0.244553096
AC242426.2	1.445742305	1.071310723	1.951040689	0.015935877
AC099508.2	0.763602355	0.59941065	0.972769763	0.029001359
AC026740.1	1.029760661	0.983601566	1.07808594	0.210085587
AC022784.1	1.018037825	0.988114438	1.048867392	0.240213826
LINC01006	1.059604333	0.943334806	1.190204512	0.328923126
SNHG3	1.05193912	1.019203463	1.08572621	0.001693899
AC004918.5	1.138200919	0.93223289	1.389675634	0.203735386
AL355574.1	1.258628674	1.027219563	1.542168973	0.02648384
SCAMP1-AS1	1.133299285	0.97899834	1.311919761	0.093792224
AL512598.1	1.089121494	0.966199724	1.227681607	0.162349345
DLGAP1-AS1	1.07953559	1.010430405	1.153367005	0.023366683
MIR4435-2HG	1.070396789	0.980014557	1.169114558	0.130676117
AP002360.2	1.053675652	1.006244688	1.103342351	0.026090121
AC013275.1	0.968546859	0.888516605	1.055785579	0.467664364
LINC00847	1.064623718	0.994458058	1.139740034	0.071828377
VPS9D1-AS1	1.123680176	1.019764859	1.238184594	0.018508633
LINC02041	0.990055124	0.932237764	1.051458314	0.74476565
PRRT3-AS1	1.175259913	1.083471144	1.27482478	9.93E-05
AC010883.1	1.073006176	0.947680317	1.214905738	0.266157515
PSORS1C3	1.021781537	0.875147153	1.192985096	0.785140809
AC106820.3	1.155604191	1.018465755	1.311208589	0.024842087
AC136475.2	0.99341336	0.85965594	1.14798265	0.9286336
AC026471.4	1.040976953	0.902942563	1.200112898	0.580052071
AC016773.2	1.4180481	1.095618739	1.835365115	0.007959183
LINC02604	1.102618402	0.953482674	1.275080684	0.187664141
AC007038.1	1.309920724	1.019786554	1.682599457	0.034571338
AC010326.3	1.095208246	1.014935531	1.181829847	0.019196731
AC243964.3	1.016199429	0.980378542	1.053329132	0.380127513
ARRDC1-AS1	1.057771652	0.961484975	1.163700833	0.248750902
MMP25-AS1	0.976698551	0.751007225	1.270214224	0.860400827
GAS5	1.008080919	1.003568034	1.012614098	0.00043839
LINC01186	1.123405085	0.926655204	1.361929422	0.236197606
AC132192.2	1.214959443	1.045678189	1.411645058	0.010977653
AC007405.4	1.05315583	0.955844297	1.160374348	0.295096114
HCP5	0.97484229	0.949939762	1.000397634	0.05362509
AP002761.4	1.064318309	0.893653926	1.267575099	0.484522256
AC009779.3	1.065027897	0.97841071	1.159313171	0.14548408
IDH1-AS1	1.185333174	1.019793808	1.377743935	0.026735983
AL162595.1	0.842774684	0.610764966	1.162917337	0.29776894
LINC00853	1.047381904	0.968489226	1.132701142	0.24660843
LENG8-AS1	1.197752769	0.988157674	1.451804437	0.065976697
AC008443.5	1.170545361	1.064576965	1.287061891	0.001144031

AC005261.3	1.153084584	1.036950262	1.282225489	0.008541682
AC022306.2	1.20782334	1.014555094	1.437908331	0.033805159
AC142472.1	1.246586365	0.963945575	1.612100938	0.092945049
SNHG21	1.390705754	1.017016299	1.901702555	0.038862276
AC011445.2	1.029295559	1.010088767	1.048867567	0.002660544
AC005840.4	0.984885935	0.711782859	1.362775588	0.926766795
NEAT1	1.003331558	0.988562077	1.0183217	0.660242724
AC016394.3	1.374524197	1.161830302	1.626155528	0.000208296
AC012313.9	0.946399517	0.833949949	1.074011751	0.393316672
AC103706.1	1.155401852	1.043422612	1.27939861	0.005482915
ARHGAP27P1	1.2962629	1.016632263	1.652807576	0.036347651
AC068580.3	1.176980664	1.016927261	1.36222475	0.028885658
AL590666.2	0.993121464	0.926139206	1.064948158	0.846382774
PRR34-AS1	1.049735995	1.010922228	1.090039995	0.011567216
ST7-AS1	1.182805286	0.797628117	1.753985741	0.403625031
TBX2-AS1	1.011040208	0.859465215	1.189346916	0.894595778
AC005332.5	1.188282507	1.06728922	1.322992203	0.001640927
AL359504.1	1.387876368	1.079909267	1.783669121	0.0104518
LINC02035	0.968019541	0.726738157	1.289407778	0.824149825
LINC02027	1.046243069	0.97718646	1.120179828	0.194440473
AP003486.1	0.836483512	0.616782429	1.134443253	0.250746559
AC012467.2	1.560983115	1.179406342	2.066012535	0.001847159
AC006026.3	1.375028547	1.082251982	1.747008586	0.009133564
AC010973.2	1.548796042	1.124758888	2.132696354	0.007356425
ITGB1-DT	1.0125959	0.991217757	1.034435117	0.250253576
AL365181.3	1.016863538	0.983197164	1.051682706	0.330304593
AC011700.1	0.994264568	0.737418233	1.340571722	0.969908129
AC008735.2	1.113210327	1.013856489	1.222300439	0.02454602
AC068506.1	1.135387165	1.009168429	1.277392333	0.034707622
AC011477.2	1.024358438	0.968798686	1.083104493	0.397629431
AL121845.4	1.036425016	0.988497879	1.08667589	0.138591515
U91328.1	1.048698163	0.855291793	1.285839344	0.647563837
AC005332.3	1.121138642	1.071482532	1.173095983	7.53E-07
AL050341.2	1.05670134	0.961341646	1.161520181	0.253064791
AC005261.1	1.066053911	0.997955262	1.138799489	0.057539461
AC139100.2	1.226954063	1.028291398	1.46399773	0.023236224
AC012615.1	1.156764751	0.979744047	1.365769655	0.085709342
ASB16-AS1	1.038931553	0.932614216	1.157368988	0.488061176
AL109615.3	0.971454508	0.88309295	1.068657451	0.551698867
LINC01549	0.994104704	0.967373292	1.021574784	0.670727677
AC087741.2	1.202957816	1.077980914	1.342424054	0.000961215
ZNF687-AS1	0.986119053	0.901956546	1.078134851	0.758766182
AC007566.1	1.02827308	0.774739883	1.364774875	0.846944224
Z98257.1	1.108214543	1.035067231	1.186531112	0.003185365

AJ009632.2	0.99445883	0.945060083	1.046439673	0.830740618
CEBPA-DT	1.06993896	1.031022224	1.110324639	0.000348787
AC026369.2	1.212560332	0.985633264	1.491733906	0.068290884
AL162411.1	1.071218507	1.014874405	1.130690736	0.012576293
AC008608.2	1.164929974	1.05070969	1.291566888	0.003738081
ARHGAP5-AS	1.43454006	1.113478105	1.848177503	0.005246483
AC010719.1	1.058065608	0.967656986	1.156921148	0.215521592
AL441992.2	1.099161673	1.02208834	1.182046929	0.010803945
LINC00205	1.210459154	1.082069834	1.354082073	0.000841603
TRIM52-AS1	1.108866522	1.055270217	1.16518494	4.35E-05
AC064807.4	0.999450702	0.764086178	1.307315501	0.996800151
AC078993.1	0.982586927	0.920152581	1.049257579	0.599967115
AP000240.1	1.535735017	1.299206832	1.815324537	4.97E-07
SH3BP5-AS1	1.170489724	0.95677596	1.431940446	0.125916973
AC023509.3	1.061528414	0.96786026	1.164261641	0.205205925
AC002456.1	1.089823644	0.976177201	1.216700794	0.125806319
AC127024.5	1.2496272	1.065503769	1.465567916	0.006141645
AC015912.3	1.177556395	1.082627612	1.280808885	0.00013825
AC099850.4	1.038497459	0.979172597	1.101416619	0.208151696
AC244090.1	1.016860773	0.994242428	1.03999367	0.145157647
AC084018.1	1.151387517	0.967639091	1.370028585	0.112031758
LINC01093	0.995305707	0.981284224	1.009527542	0.515681
AC010531.6	1.247858002	1.032227171	1.508533815	0.022157166
ZNNT1	1.113595068	1.004988754	1.233938161	0.039877934
AC110285.2	0.987255654	0.936481258	1.04078295	0.633987734
AL365330.1	1.120607178	0.876451342	1.432778281	0.363778986
ELFN1-AS1	1.047630298	1.005355252	1.091683004	0.026821421
AC008736.1	0.975249006	0.908964811	1.046366825	0.485250422
AL031985.3	1.646094461	1.219497698	2.221920532	0.001127736
AC109322.1	1.229616893	1.069931375	1.413135216	0.003587357
AL135999.1	1.013169376	0.766323601	1.339528345	0.926830414
AL365203.2	1.070136171	1.002150878	1.142733543	0.04295767
LINC00997	1.171512314	0.916298393	1.497810223	0.206701282
AC010280.2	0.959735797	0.879112519	1.047753023	0.358624342
AC084125.4	1.247263513	0.953403454	1.631697748	0.106991637
AC253536.6	0.984900273	0.9179101	1.056779469	0.672045468
AC007996.1	1.113701098	0.87615262	1.415655341	0.378970341
LINC02241	1.0169416	0.922324307	1.121265274	0.735993228
U62317.2	1.082054535	1.040885073	1.124852346	6.76E-05
HAGLR	1.023055856	0.978733604	1.069385256	0.313114248
CTBP1-DT	1.059333238	0.880731454	1.274153323	0.540642808
AC005696.1	0.946578592	0.776045332	1.154585942	0.588026653
MAFG-DT	1.122809221	1.034411457	1.218761199	0.005629425
AC015871.3	1.160827161	0.870061669	1.548763434	0.310691306

AC023090.1	1.110053307	1.017825401	1.210638233	0.018314485
AC007541.1	1.18809839	0.974495654	1.448521376	0.088293258
AC064836.2	1.242924221	1.046414362	1.476337363	0.013261732
AC007406.2	0.992520638	0.920958759	1.069643138	0.844116554
AC095057.3	1.31062449	1.047391444	1.640013926	0.018042623
LUCAT1	1.231653617	1.096240364	1.383793811	0.000454521
ZFAS1	1.008381074	1.00241295	1.014384731	0.005856589
SNHG30	1.091875867	1.030715551	1.156665298	0.002802476
LINC01703	1.138847513	0.988784384	1.311685014	0.071308285
AC004816.1	1.165685072	1.045468375	1.299725291	0.005768656
AC004148.1	1.263907726	0.996364249	1.603291911	0.053613076
WNT5A-AS1	0.96138396	0.778799022	1.186774884	0.714014693
AC024075.2	1.091253445	0.972240598	1.224834762	0.138298997
AL117379.1	1.311660185	1.025657444	1.677414278	0.030630519
AC084824.5	1.247683532	1.005785384	1.547759812	0.044175119
AC002398.1	1.206414871	1.060283734	1.372686191	0.004392065
AC048341.2	1.111016513	0.99126504	1.245234767	0.070421271
AL133353.1	1.118995866	0.941559162	1.329870493	0.201830715
AC016747.1	1.086299689	0.970525981	1.215884003	0.14996659
AC104958.2	1.008997838	1.000486616	1.017581466	0.038216474
YTHDF3-AS1	1.032514442	0.930118795	1.146182701	0.548193389
AL390728.6	1.052500485	1.022938694	1.082916579	0.00043115
DSCR8	1.009491943	0.963014828	1.058212141	0.694435384
LINC00623	1.343607794	1.129892186	1.597747047	0.000832869
LINC01770	1.015411366	0.947067403	1.088687289	0.667055401
AC090587.1	1.187783223	0.994162182	1.419113512	0.058029527
BACE1-AS	1.182504364	1.08699014	1.286411459	9.58E-05
AP003119.1	1.171959688	1.013151727	1.355660236	0.032692826
SNHG25	1.021572333	1.00903433	1.03426613	0.000705627
AC120053.1	1.149278477	1.017087291	1.298650595	0.025632761
LINC00342	1.124871988	1.005362596	1.258587692	0.040044509
AL603839.3	1.250300592	0.993405238	1.573629282	0.05696488
AL359921.2	1.22575104	1.081114586	1.38973762	0.001486027
PRANCR	1.344655871	1.060882454	1.704335296	0.014339394
LINC02062	1.302291439	0.829060692	2.045643954	0.251651169
ANKRD10-IT1	1.05561774	0.998211102	1.116325806	0.057800991
AC011477.1	1.063112457	0.988859393	1.142941153	0.097580695
AC084036.1	1.024876415	0.969719135	1.083171021	0.383992508
AC083843.3	1.18488512	0.975961911	1.438532316	0.086507713
AFDN-DT	1.300425583	1.011159835	1.672442513	0.040715085
AC131009.3	1.152679045	1.036562139	1.281803503	0.008720537
LMNTD2-AS1	1.080520972	0.923317486	1.264489831	0.334342422
AC090015.1	1.056669326	0.950446832	1.174763307	0.307849321
AL360181.1	0.98741656	0.945241248	1.031473675	0.569640766

SNHG20	1.331988721	1.134556287	1.563777817	0.00046137
AC092171.5	1.084047247	0.967922818	1.214103451	0.162717033
LINC01270	0.986584941	0.821781078	1.18443935	0.884846708
AC112206.2	0.981588961	0.915016119	1.053005371	0.604044568
AL121944.2	1.038366381	0.934841179	1.153356062	0.482317613
LINC01836	1.012371524	0.923765467	1.109476527	0.792465592
AC055822.1	1.511569446	1.14036413	2.003607558	0.004059448
AC009005.1	1.171179731	1.074283894	1.27681516	0.000335494
TMEM161B-A	1.252440179	1.015725861	1.544320631	0.035208851
AC010969.2	1.557394813	1.249872086	1.940581465	7.90E-05
LINC01503	1.056676588	1.01808561	1.096730373	0.003681796
AL132989.1	0.976409141	0.750206176	1.270817064	0.859073538
LINC01124	0.986830568	0.945025436	1.030485034	0.548334236
AC092171.2	1.051969831	0.986064434	1.122280134	0.124825527
PXN-AS1	1.245491713	1.07035898	1.449279761	0.004519895
SNHG6	1.006938413	1.003252148	1.010638224	0.000219801
AC007448.4	1.084775044	0.8942453	1.315899447	0.408958725
ELF3-AS1	1.051904004	0.985461481	1.122826266	0.128502377
LINC01138	1.258674993	1.080402646	1.466363254	0.003153083
AC010735.2	1.059455055	0.884885878	1.26846302	0.529549653
COA6-AS1	1.101927835	1.032901371	1.175567182	0.003274055
AC021078.1	1.340695442	1.004651384	1.789142281	0.04642756
NIFK-AS1	1.188734444	0.99763587	1.416438223	0.053175419
LINC01287	1.00330851	0.981820396	1.025266913	0.764921457
AC026401.3	1.0791255	1.03424947	1.125948699	0.000441564
TYMSOS	1.109949798	1.04612213	1.17767182	0.000556111
PTOV1-AS1	1.203643436	1.017470535	1.423881548	0.03061949
TMEM44-AS1	1.02729264	0.993828805	1.061883257	0.11102473
UBR5-AS1	1.359322294	1.152754805	1.602905571	0.000261954
PRKAR1B-AS	1.071922379	0.953027609	1.205649842	0.246911522
MALAT1	1.040119472	1.004062944	1.077470813	0.028873006
SLC25A25-AS	1.065715474	0.977900711	1.161415939	0.146883442
LINC01151	1.014475365	0.973732775	1.056922692	0.491964288
AC068580.1	1.195141117	1.022387999	1.397084365	0.025226143
AC009065.5	1.343060417	0.96302597	1.873066084	0.082216375
NUP50-DT	1.039278043	0.978517018	1.103812025	0.210053712
Z95115.1	1.172840716	1.027888156	1.338234455	0.017854369
AC104794.3	1.027353876	0.960761507	1.098561901	0.429961868
AC008549.1	0.995120367	0.984830186	1.005518066	0.356347437
SLC6A1-AS1	1.026362612	0.794379077	1.326092595	0.842221447
AC007298.2	0.945860726	0.756535688	1.18256485	0.625236469
DNAJC3-DT	1.139852717	0.934789289	1.389900624	0.195814736
AC018904.1	1.047639298	0.970563713	1.130835703	0.232616937
AL049840.6	1.079147731	1.025796321	1.135273934	0.003234684

AC009237.15	1.064952163	0.841177436	1.348256696	0.601051751
AL021807.1	1.257308445	1.040943453	1.51864592	0.017480945
AC124798.1	1.07065026	0.918767247	1.247641319	0.381807964
HCG18	1.124392183	0.857176475	1.474909563	0.397088807
NRAV	1.135894038	1.01585698	1.270115075	0.02534854
LINC02506	1.036357264	0.999657628	1.074404224	0.052215962
AC040970.1	1.272569314	1.088696423	1.487496996	0.002467771
PITPNA-AS1	1.028434255	0.965131684	1.095888815	0.387033781
AL162582.1	0.981648153	0.943004473	1.021875425	0.366038642
